# The Potential to Leverage Real-World Data for Pediatric Clinical Trials: A Proof-of-Concept Study

**DOI:** 10.2196/72573

**Published:** 2025-05-30

**Authors:** Jens Declerck, Joanne Lee, Anando Sen, Avril Palmeri, Rianne Oostenbrink, Viviana Giannuzzi, Simon Woodworth, Salma Malik, Fenna Mahler, Beate Aurich, Rebecca Leary, Dipak Kalra, Volker Straub

**Affiliations:** 1 The European Institute for Innovation Through Health Data Ghent Belgium; 2 John Walton Muscular Dystrophy Research Centre, Translational and Clinical Research Institute, Newcastle University and Newcastle Hospitals NHS Foundation Trust Newcastle upon Tyne United Kingdom; 3 Erasmus Medical Center Rotterdam The Netherlands; 4 Fondazione per la Ricerca Farmacologica Gianni Benzi Onlus Bari Italy; 5 INFANT Research Centre University College Cork Cork Ireland; 6 European Clinical Research Infrastructure Network Paris France; 7 Department of Pharmacy Division Pharmacology and Toxicology Radboud University Medical Center Nijmegen France; 8 Drug Safety Consulting SASU Paris France

**Keywords:** electronic health records, observational research, safety studies, data interoperability, rare diseases, data collection

## Abstract

**Background:**

Pediatric clinical research, especially in rare diseases, faces persistent challenges including the identification and recruitment of eligible patients, assessing protocol feasibility, and ensuring efficient trial execution. These issues are compounded by small, age-stratified populations and fragmented clinical data. Real-world data (RWD), especially when drawn from electronic health records (EHRs), present an opportunity to support innovative trial designs, such as real-world comparator arms and postmarketing surveillance. However, realizing this potential depends on the routine availability of structured, reusable clinical data.

**Objective:**

This proof-of-concept study aimed to assess the availability and structure of routine clinical data in European pediatric hospitals, focusing on data elements relevant for use in comparator arms and postmarketing surveillance studies. The study focused on 2 disease areas—neurofibromatosis (NF) and atopic dermatitis (AD)—as examples of rare and common conditions in children, respectively.

**Methods:**

An inventory of 113 high-value clinical data items was developed based on expert analysis of clinical protocols for NF, AD, and safety studies. These items were included in a structured web-based survey disseminated through the connect4children (c4c) National Hub network, reaching sites across. Europe. Respondents were asked to indicate how each data item is collected and stored: in structured/coded EHR fields, as free text, in external systems, or on paper.

**Results:**

Survey responses from 24 hospitals across 11 European countries revealed considerable variability in how data are captured and stored. While many general clinical and drug safety data elements—such as demographics, vital signs, and medication use—were often collected in structured formats, disease-specific and contextual variables were frequently captured as free text or not documented in a standardized way. For example, structured data capture was more prevalent for basic demographic and safety-related variables, whereas only a minority of sites recorded key disease-specific clinical details in a structured form. Lifestyle and family history data were among the least consistently documented. These gaps in structured data entry reduce the immediate reusability of EHR data for secondary research purposes.

**Conclusions:**

This study highlights gaps in the structured documentation of pediatric clinical data across European sites. While the routine collection of many variables is promising, the lack of structured and coded formats poses a barrier to reusing these data for observational studies or comparator arms. As a first step toward the broader integration of RWD into pediatric research, this study demonstrates the feasibility of assessing EHR data availability and sets the stage for future scaling across more diseases and sites.

## Introduction

A major challenge to the conduct of pediatric clinical trials is finding eligible patients in sufficient numbers to make a proposed trial viable. Further challenges exist in trial planning and feasibility. This is especially the case in rare disease research, which presents unique challenges in the context of pediatric studies.

The classical patient recruitment pipeline includes sending candidate eligibility criteria to the sites of known investigators in a disease area for them to estimate the number of likely eligible patients. This helps confirm that the criteria will lead to a viable trial (protocol feasibility) and indicates the likely distribution of recruitable patients and therefore which sites might be most appropriate to include in the study (site selection).

Today, real-world data (RWD) are recognized as an important source of information not only for public health purposes but also for scientific health research. As an alternative or complementary to the traditional clinical research setting, such as clinical trials, evidence generated in the real world contributes to a better understanding of diseases and the life cycle of medicines. RWD collected both retrospectively and prospectively and included in electronic health records (EHRs), registries, claims, and prescription data can provide a wide spectrum of evidence [1]. The collection of health data represents an opportunity to gather evidence in a field where data are scarce, heterogeneous, and dispersed across countries—factors that make traditional clinical research difficult and lengthy.

Despite the advanced adoption of EHR systems, many of these patient estimates are made based on expert judgment and simplistic chart reviews and are therefore prone to errors [2]. They can be over or underestimated, and there might be patients who are eligible but who were not identified during recruitment and so are never invited to participate.

The drawback of launching a study that might not recruit enough patients can bring many challenges, the study may fail or face significant delays—resulting in further delays in treatment access for children. Rare disease populations present a particular challenge in terms of trial recruitment, with 32% of trials citing the lack of patient accrual as the reason for trial noncompletion [3]. Alternatively, the trial protocol may need to be amended to modify those criteria and enlarge the candidate pool of eligible patients and sites, which carries a significant expense and is more challenging with small populations such as pediatric patients [4,5]. This is also impractical in pediatric studies, which tend to have smaller, age-stratified populations, especially in rare diseases. Some sites might rarely or never recruit a patient for a study, which also presents an avoidable cost in site preparation.

There has been important multinational research over the past 15 years that demonstrated the added value of utilizing hospital EHRs to predict eligible patient numbers more accurately, with consequent trial efficiency and cost benefits, without prejudicing the confidentiality of the patient data within each EHR system [6,7]. Technology products based on this research are now on the market and scaling up their deployment networks, as in the study by Palchuk et al [8].

EHR data may be useful for different kinds of clinical study, including (1) classical 2-armed trials comprising an intervention arm and a control arm both recruited prospectively, (2) generating comparator arms from a nonrecruited but observed comparable population that can serve as a control group in trials where patient populations are scarce, (3) postmarketing surveillance or safety studies for the long-term effects of existing drugs on the market, and (4) having a potential recruitment pool for future clinical trials.

Not all clinical trials are interventional in that they involve the administration of a novel medicine or clinical procedure that also introduces a placebo effect. Some are observational, utilizing longitudinal data to study the course of a disease and the effectiveness of different existing standards of care and care pathways. Observational studies still rely on a similar case-finding methodology but aim to access longitudinal (historical) data as well as follow up with patients prospectively without interfering in their treatment. Another type of noninterventional research involves conducting clinical safety studies, which monitor the occurrence of adverse events in patients taking existing treatments, including new treatments, to better understand the prevalence and profile of adverse events and side effects. EHRs can be an important source for providing longitudinal data as well as a log for adverse events.

Because of the rarity of eligible patients, particularly pediatric patients, the second model, which uses a real-world control arm, is attractive. It requires active patient recruitment only for the interventional arm of a clinical study, while the control arm can be drawn from a population of patients identified through EHRs. Patient control groups are usually case-matched (ie, matched pairs) to correspond to the intervention arm patients using the same eligibility criteria [9-12].

The research to demonstrate the value of using hospital EHRs to improve clinical trial design and conduct has almost all been based on work undertaken in adult clinical trials and, therefore, on finding adult patients [13]. There is a lack of research at an equivalent scale to adult studies to verify the reuse of hospital EHRs for pediatric clinical trials. The European Union Patient-Centric Clinical Trial Platforms (EU-PEARL) project [14], funded through the European Commission Innovative Medicines Initiative (IMI2) program, has examined the feasibility of this undertaking as well as practical approaches that could be taken to establish longitudinal observational cohorts in 4 disease areas, including neurofibromatosis (NF), most often studied in children [15]. Its primary objective was to develop and validate a methodology for conducting multiarm platform trials. Platform trials are disease-specific, open-ended trials where new interventions can be added and old ones removed over time. The treatment groups do not have to be specified at the start of the trial [16]. Platform trials may contain a large observational cohort fulfilling a set of eligibility criteria documented within a master protocol for that disease area. As part of its method, the EU-PEARL has designed observational clinical study protocols to determine the longitudinal data that would be of greatest value to prioritize collection [9,17].

Given the limited resources and time available for this research, it was not possible to investigate the other important topic of the feasibility of utilizing EHRs to establish a recruitment pool (longitudinal cohort) of potentially eligible patients for future trials. Calculating patient numbers for such a pool would require queries on EHR data, similar to those conducted for recruitment into an interventional study. This would require a combination of ethics approvals, consent, data protection, and information security measures on top of a technology capability that was beyond the scope of this investigation. Instead, this study focused on answering a more foundational research question: To what extent do specialist centers treating pediatric patients routinely collect the types of clinical data—particularly in structured and coded formats—that could be reused for secondary research purposes? Specifically, we sought to assess the availability and structure of routinely collected data that could be repurposed to support pediatric clinical research, particularly for (1) constructing real-world comparator arms and (2) informing safety studies.

The research was undertaken as part of the conect4children (c4c) project. c4c is funded through the European Commission Innovative Medicines Initiative (IMI2) program and has the vision to create “Better Medicines for Babies, Children, and Young People Through a Pan-European Clinical Trial Network.” One strand of c4c work has focused on the standardization, interoperability, and reusability of pediatric data. Previous tasks within this strand have focused on standardization of pediatric clinical trial data [18,19], enabling pediatric data to be findable, accessible, interoperable, and reusable [20]; fostering collaboration through global networks [21]; and ensuring the European Health Data Space (EHDS) addresses pediatric data concerns [22]. This study builds on the previous work by addressing the reuse of RWD.

## Methods

The work to develop the use cases and to develop the inventory of data items (steps 1 through 4) started in June 2022 and took until October 2023 to formulate the site survey instrument. The site recruitment and onboarding (step 5) took place from January to March 2024, and the survey itself ran until April 2024.

### Confirm the Clinical Research Use Cases (Study Types) to Focus on

The research team in consultation with the c4c project leadership opted to examine the availability of routinely collected EHR data that could be used as longitudinal observational data for either a control arm population or a clinical safety study.

This investigation therefore sought to establish whether the EHR systems, at numerous clinical sites across Europe, had the capability to capture the data items that would ideally be needed for these 2 study types, preferably as data in a structured and coded form. It was not possible to examine the records of actual patients, even in aggregated or anonymized form, to establish frequency distributions of data item values or to estimate patient numbers. Therefore, General Data Protection Regulation (GDPR) principles did not apply to this research.

### Confirm the Example Disease Areas to Focus on

NF, a rare genetic disorder, and atopic dermatitis (AD), a common inflammatory skin condition, were selected to reflect the spectrum of pediatric disease prevalence and complexity. NF typically involves longitudinal specialist care, multisystem evaluations, and complex diagnostic data, while AD is more commonly managed across general pediatric settings with routine clinical workflows. This contrast allows us to assess EHR data capture feasibility across both rare and common conditions, each with differing documentation practices and data requirements. By including both extremes, we aimed to identify patterns that may apply more broadly across pediatric diseases.

### Review of Study Protocols and Eligibility Criteria for RWD Relevance

A team of NF experts from both contributing projects, c4c and EU-PEARL, along with experts from Sanofi and the University of Cork, examined the NF master protocol developed in EU-PEARL [9] and extracted the data items that were cited in the protocol either as eligibility criteria or as important data items to routinely collect. From these data items, a data item inventory was derived for this investigation. The initial inventory was not filtered or prioritized but extracted in its entirety and comprised 70 data items. A similar process was followed for AD, which included a review of eligibility criteria from 24 Novartis trials as well as 2 Dupilumab trials (NCT03054428 and NCT03345914) along with inputs from the University of Cork. A total of 186 unique data items were extracted.

Because there was no equivalent prior master protocol for clinical safety studies, 2 c4c investigators working within Novartis retrieved a portfolio of their recent nonconfidential clinical safety study protocols (across multiple therapeutic areas) and similarly extracted the data items that were common between these protocols, in effect replicating the methodology that was used by the NF team during EU-PEARL. This initial inventory was also not filtered or prioritized but extracted in its entirety and comprised 92 data items.

Each list was initially kept separate, and in each case, 3 clinicians and 2 patients were asked to independently review the data element inventory to mark the ones that they believed would be most relevant to be collected during clinical encounters or would be used for an RWD study. In practice, the experts largely agreed with each other and deprioritized very few data items. In retrospect, it is likely that this review step could have been avoided.

### Consolidate as a High-Value Data Set Across Use Cases and Convert Into a Web-Based Survey

The inventories were then combined and deduplicated. Inevitably, several data items relating to demographics, family history, laboratory and radiology investigations overlapped with each other, although their values could differ. For example, both inventories sought family history but one looked for a family history of allergic or atopic conditions, while the other looked for a family history of neurological conditions.

The process of deduplicating and combining data items was undertaken through several iterations by the work package experts in both conditions, as well as general clinical research and informatics experts through teleconference workshops.

The final inventory of 113 data elements combining NF and AD comprised the following items: demographic and diagnosis items (n=6, 5.3%), NF-specific features (n=12, 10.1%), AD-specific features (n=14, 12.3%), drug and vaccine safety features (n=30, 26.5%), relevant family conditions (n=6, 5.3%), instances of the presence of other conditions, including in the mother (n=7, 6.2%), lifestyle information (n=4, 3.5%), allergy information (n=8, 7.1%), examination findings (n=12, 10.1%), and encounter history (n=14, 12.3%).

The data inventory was incorporated within a web-based survey, preceded by an introductory landing page including instructions for completing the survey and how confidentiality would be handled. The next screen captured basic information from the respondent about the type of health care organization, its country, and the EHR system in use.

The heart of the survey was structured to gather data across a range of relevant data item categories. In total, 20 different categories were included, ensuring the collection of both general and disease-specific data items. Of these, 10 (50%) categories were general and covered data applicable to both disease areas, such as demographics, allergies, and other general health indicators. These general conditions were designed to capture essential patient information that is relevant across multiple conditions. The remaining 10 (50%) categories were disease-specific, with a focus on data specific to either AD or NF. This distinction ensured that condition-specific details were captured while maintaining a consistent core set of shared information across both diseases. The complete survey structure, along with the full list of categories, is provided in Multimedia Appendix 1.

For each data item selected for inclusion in the survey, sites were asked whether they collect the specified item within their EHR system. The aim was not only to determine whether the data were collected but also to understand how they were stored and managed. Sites were presented with several response options to describe the method of data collection, ranging from highly structured electronic formats to traditional paper-based methods. Textbox 1 outlines the different methods of data collection as reported by sites.

**Different methods of data collection as reported by sites.**

- Within the electronic health record (EHR) system, in a structured and coded format
- Within the EHR system as free text
- Within a separate electronic system, not linked to the EHR
- On paper charts or files
- Not directly collected but could be derived from other EHR data

In addition to these options, sites had the option to indicate if the data item was not collected at all or if they were unable to answer the question.

The survey was designed and deployed using LimeSurvey, a flexible platform that facilitates the collection of survey data. LimeSurvey was selected for its ability to handle various question types, manage responses from many sites, and monitor participation in real time.

### Verify Data Capture at Clinical Sites and Survey EHR Data Dictionaries

To deliver high-quality pediatric clinical trials, c4c has established a network of National Hubs (NHs) and qualified hospital sites across Europe with pediatric expertise [23]. This network includes 20 NHs covering 21 countries (with the Finnish NH overseeing both Finland and Iceland), encompassing approximately 270 hospital sites eligible to participate in this study. The survey was disseminated through the NHs, with each hub responsible for selecting and forwarding it to relevant local sites within their network. There were no formal inclusion or exclusion criteria applied at the site level, and participation was based on each site’s interest and willingness to contribute. Given the large number of sites and the diversity of EHR systems in use, the NHs served as an effective and practical mechanism for targeted survey distribution across multiple countries.

The survey proposal was introduced to NH representatives at the c4c General Assembly in June 2023. Before the meeting, an invitation was sent to all NH representatives to invite them to attend this meeting and give feedback on the survey proposal. Representatives from 3 NHs attended this meeting and provided feedback on the proposal and suggestions on how to increase the number of responses. The feedback suggested that further input was needed from NH and the research would need to highlight what the incentives were for NH completing the survey. The main takeaway from this discussion was the need to clearly explain to the NHs the benefits of using data collected in EHRs to support pediatric clinical trials, should it be deemed feasible. It was also agreed in this meeting that completing this survey would require multistakeholder collaboration.

**Figure 1:**
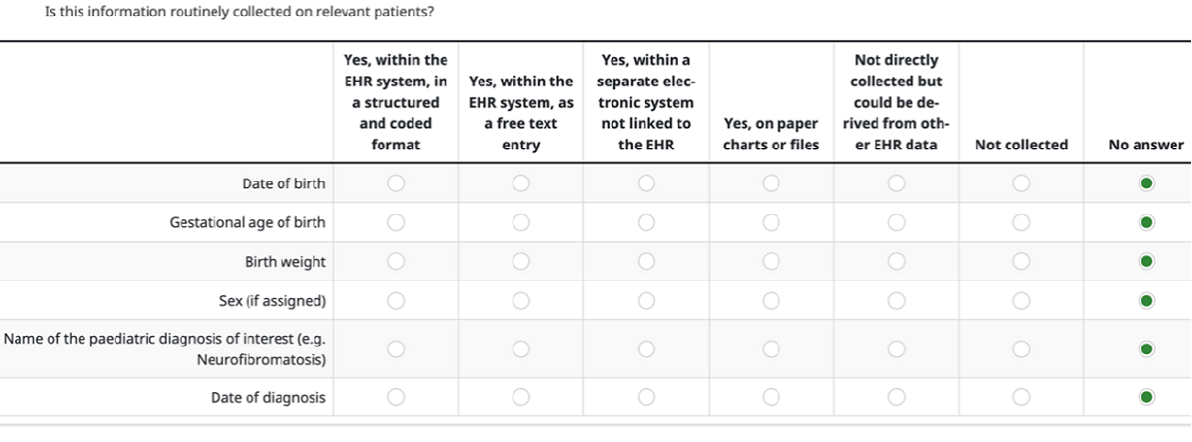
Example screenshot of the web-based data item survey. EHR: electronic health record.

The survey needed to have options for multiple people (eg, clinicians and IT managers) to access and contribute to the same survey response. This would help ensure that answers were well-informed and of high quality. The survey could be partially completed, saved, and shared with other team members to enable multistakeholder completion (Figure 1). As such, the survey did not include a specific question about the role of the respondent since it was expected that answers would reflect input from both clinical and technical stakeholders. The final responses received were therefore the result of collaborative input from a mix of roles. In most cases, clinical specialists provided input on routine care practices and variable relevance, while technical or IT personnel contributed information about EHR system configuration and data structure. Although specific job titles were not collected, the level of system-specific detail in many responses indicates cross-functional team involvement at each site.

Once the draft survey was compiled, it was sent to NH representatives from Belgium, Germany, and Ireland for review, and minor changes to the layout and wording to improve its clarity were made based on their suggestions. During an annual NH Forum meeting, the survey proposal was introduced to a larger audience of NH representatives. It was agreed during this meeting that the survey and an accompanying letter would be sent to NH coordinators, who would then distribute the survey to relevant sites based on their knowledge of each local site. Another action taken to increase the chances of a high response rate was to include both a short and long read in the letter, explaining the purpose of the survey.

The working group organizers contacted the c4c Single Point of Contact (SPoC) and asked them to circulate the letter and link to the survey. The SPoC provides 1 email address and 1 web-based form to collect requests to c4c NHs and cascades those requests to all NHs.

At the conclusion of the survey, the data were extracted from LimeSurvey for further analysis. The platform’s export functionality enabled us to download the data set in CSV format. Prior to analysis, the data underwent a cleaning process, which involved removing incomplete or duplicate responses and addressing any missing data. For data analysis, the cleaned data set was imported into R software (version 4.4.1; R Foundation for Statistical Computing). The R software provided the necessary tools for both descriptive and inferential statistical analyses, enabling us to extract meaningful insights from the survey responses.

## Results

### Survey Participation and Site Characteristics

A total of 24 complete survey responses were received from hospital sites across 11 European countries. These included Austria, Belgium, Czech Republic, Estonia, Ireland, Italy, the Netherlands, Portugal, Spain, Switzerland, and 1 unspecified country. Switzerland and the Netherlands were the most represented, contributing 5 and 4 responses, respectively.

Hospitals reported using a range of EHR systems. While some employed well-known platforms such as Epic and Cerner, others used regional or in-house solutions. In 6 (25%) cases, respondents were unable to specify the EHR system in use, suggesting that some submissions may not have been completed by the IT personnel.

Most sites provided care for the full pediatric age range, including neonates, and nearly all also treated adolescents. A subset of sites additionally cared for adults, often in mixed pediatric-adult institutions.

**Table 1:** Countries and frequencies of electronic health record (EHR) systems used.

Country and EHR^a^ system		Responses, n
**Austria**		2
	PCS	1
	N/A^b^	1
**Belgium**		3
	Hix	1
	PRIMUZ	1
	COSTEC AG	1
**Czech Republic**		1
	N/A	1
**Estonia**		1
	N/A	1
**Ireland**		1
	Cerner	1
**Italy**		2
	Trackcare	1
	N/A	1
**Netherlands**		4
	Chipsoft	3
	EPIC	1
**Portugal**		2
	Oracle	1
	N/A	1
**Spain**		2
	SAP	1
	HP-HCIS	1
**Switzerland**		5
	In house	2
	EPIC	2
	COSTEC AG	1
**N/A**		1
	EPIC	1

^a^EHR: electronic health record.

^b^N/A: not applicable.

### Age Ranges of Patients

The sites were asked to specify the age ranges of patients they routinely care for. The responses confirmed a consistent pediatric focus across participating hospitals. Nearly all sites reported providing care for neonates, with only 2 exceptions—one in the Netherlands and one in Spain. All hospitals surveyed offered care for children up to 12 years of age, and the vast majority also provided services for adolescents aged 12 to 18 years, demonstrating comprehensive pediatric coverage across the network. A smaller number of hospitals also treated adult patients aged 18 and older. This adult care was typically offered within mixed pediatric-adult institutions and was most reported in Austria, Belgium, Switzerland, and selected sites in the Netherlands. Importantly, even in hospitals that included adult services, a clear emphasis on pediatric care was maintained, in line with the aims of this study.

### Disease-Specific Data Availability

**Figure 2:**
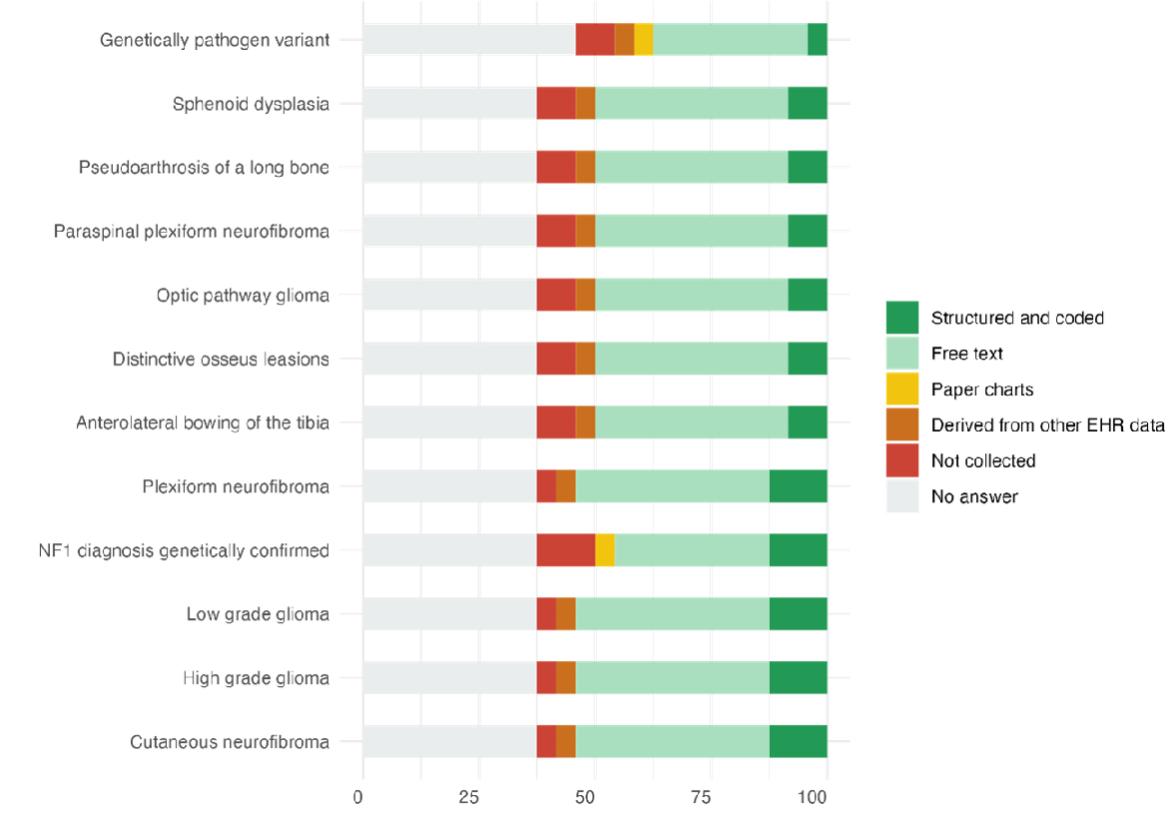
Percentage-stacked bar plot for neurofibromatosis (NF) data items. Each color represents the percentage of sites collecting the data items in a particular format. EHR: electronic health record.

Data collection for NF-specific variables varied considerably across sites. Key items such as cutaneous neurofibroma, high- and low-grade glioma, and plexiform neurofibroma were often recorded as free text, with limited use of structured and coded formats. For instance, cutaneous neurofibroma was captured in a structured format at only 13% (n=3) of sites, while 42% (n=10) recorded it as free text, making systematic data extraction more challenging. Similarly, only 13% (n=3) of sites documented confirmation of an NF-1 diagnosis in a structured way, and 38% (n=9) did not respond to this item at all, pointing to notable gaps in the documentation of genetic diagnosis. A detailed breakdown is available in Multimedia Appendix 1, and key results are summarized in Figure 2.

**Figure 3:**
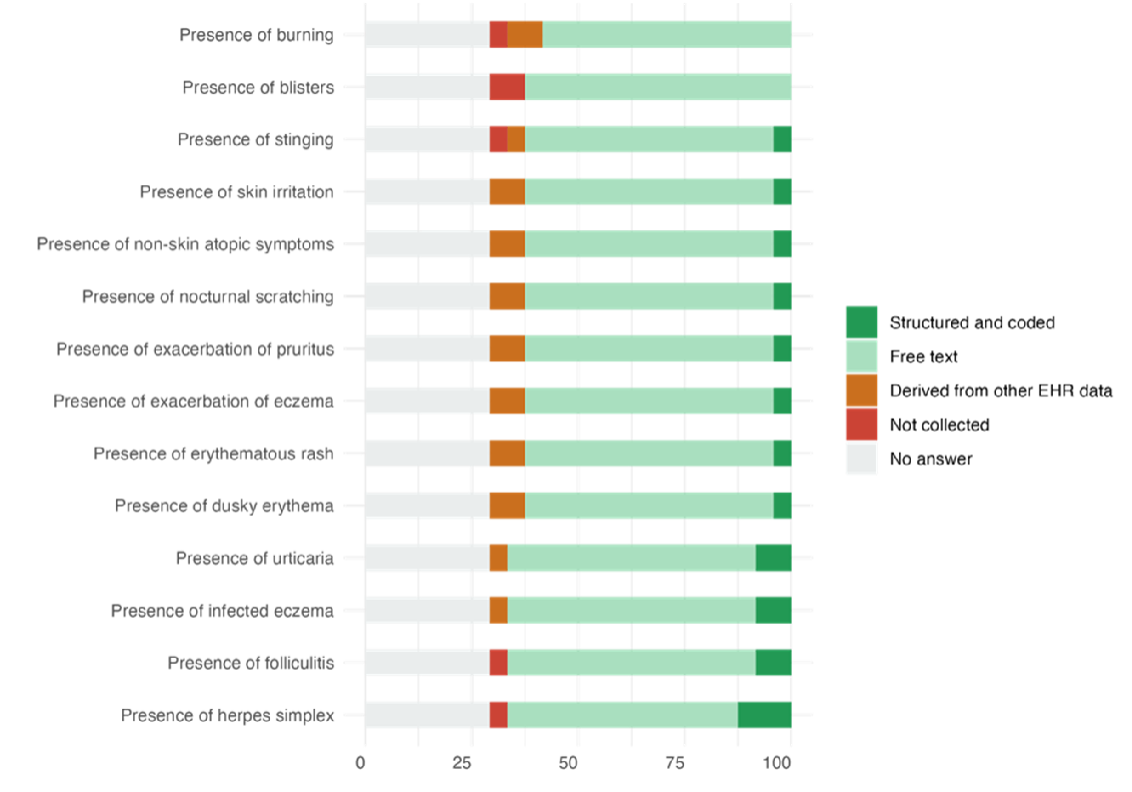
Percentage-stacked bar plot for atopic dermatitis (AD) data items. Each color represents the percentage of sites collecting the data items in a particular format. EHR: electronic health record.

The data collection for AD-related symptoms was largely unstructured across sites. Most responses indicated that symptoms such as herpes simplex and folliculitis were recorded in free text formats at 58% (n=14) and 54% (n=13) of sites, respectively. Only a small proportion of sites captured these data in structured, coded formats. In addition, a notable number of sites did not collect this information or left the corresponding survey items unanswered, suggesting potential gaps in the systematic documentation of symptomatology. This lack of structured data could hinder the ability to monitor disease progression consistently or compare patient outcomes across different settings. Other skin-related symptoms, such as infected eczema and urticaria, also showed similarly low levels of structured data capture. Further details can be found in Multimedia Appendix 1, and a summary of results is presented in Figure 3.

### Drug and Vaccine Safety

**Figure 4:**
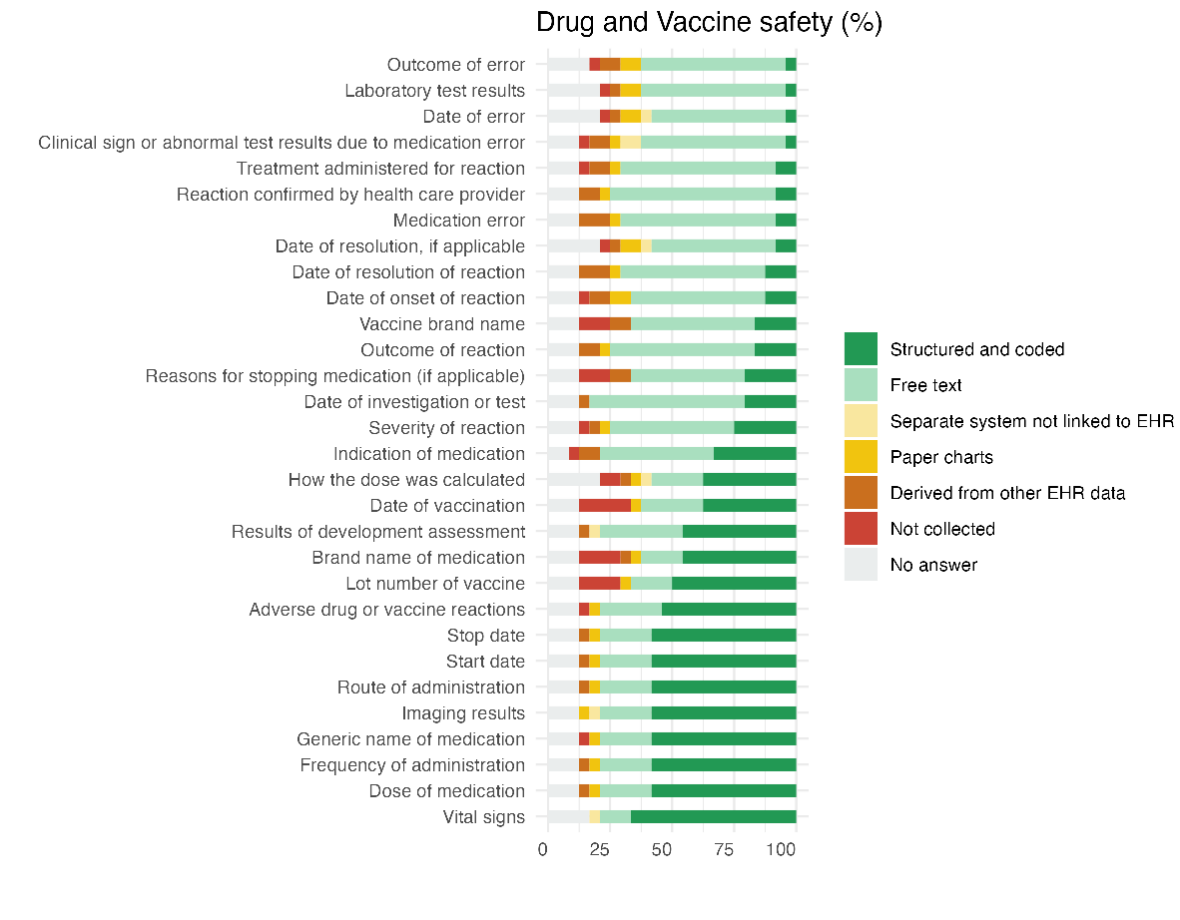
Percentage-stacked bar plot for drug and vaccine safety data items. Each color represents the percentage of sites collecting the data items in a particular format. EHR: electronic health record.

Data related to drug and vaccine safety were predominantly collected in structured and coded formats across sites, with over 50% (n=12) of responses indicating structured capture for most key variables. Vital signs (n=16, 67%), medication dose (n=14, 58%), and frequency of administration (n=14, 58%) were among the most consistently structured items. However, some fields, such as vaccine lot number (n=12, 50%) and medication brand names (n=11, 46%), were less systematically recorded. This inconsistency may hinder pharmacovigilance, particularly in the context of vaccination, where missing or unstructured data could impact safety monitoring and traceability. Further details are provided in Multimedia Appendix 1, and key findings per category are summarized in Figure 4.

### Summary of Additional Data Domains

In addition to disease-specific findings, the survey assessed a wide range of cross-cutting data domains relevant to observational studies and safety monitoring. Core clinical variables such as demographics, vital signs, and encounter history were generally well captured in structured formats across most sites. However, data related to family history, lifestyle and social context, allergy profiles, and certain procedural or therapeutic details (eg, radiotherapy and phototherapy) showed significant variability. Many of these items were recorded as free text or not collected at all, particularly in areas such as family conditions, psychiatric history, and lifestyle exposure (eg, smoking and caregiver education). Data on past procedures, allergy testing, and comorbidities were inconsistently structured, limiting their reusability. While structured formats were used for some key variables, especially within drug safety and encounter tracking, numerous gaps remain in the consistent documentation of clinical context and longitudinal patient information. A detailed breakdown of these additional data domains is provided in Multimedia Appendices 1 and 2.

## Discussion

### Principal Findings

The reuse of RWD for clinical research studies is now well established in adult patient populations in therapeutic areas such as oncology [24,25]. The technology products and the data mapping skills needed to harness the knowledge within EHR systems for the main use cases of protocol feasibility, site selection, patient recruitment, and screening are now mainstream and available commercially. Moreover, there are growing deployment networks of connected hospitals globally [26].

However, while the same technical solutions could be applied to pediatric trials in general, there is an anecdotal skepticism about whether the underlying EHR systems are adequately designed to capture the necessary information for these patient populations. Although this study included a rare disease (NF) and a common pediatric condition (AD), the observations made here are relevant to a wide range of pediatric conditions, with vaccines possibly being an exception due to their specific data requirements. The high number of responses captured as “free text” in our study suggests that EHRs may not allow for adequate structured data collection. This highlights a potential gap in their ability to collect clinically relevant information in a reusable format for research purposes.

Our intent has been to bring greater visibility to the importance and realistic plausibility of leveraging EHR data to help tackle the challenge of the limited number of pediatric patients and the difficulty of finding them when developing clinical trials. The findings of this survey suggest that data items are often available, though not always collected in a structured and coded form. It appears that administrative information may be well captured in structured systems, while more specific medical information tends to be recorded as free text. This may indicate a disparity in the training of staff responsible for inputting clinical versus administrative data or limitations in the EHR systems themselves, which may not support the structured entry of more complex medical information. These relevant data items are often available in narrative form, such as clinic letters and discharge summaries, and may be scattered across multiple hospital subsystems or subject to data entry quality and compliance with EHR maintenance protocols. This can result in data being lost, inconsistently recorded, or stored in multiple systems or formats, making it difficult to harness this information efficiently for research purposes.

In addition to highlighting the importance of scaling up the evidence and confidence in EHR-informed clinical research in children and rare diseases, we hope that health care providers and clinical research organizations will advocate for the value of structured and coded data to EHR system developers and health care organizations. The goal is to improve both the design of EHR systems and the training of staff in capturing medical information in structured and computable data formats.

Data protection regulations and informed consent remain important challenges when reusing EHR data, particularly across international borders. Legal and ethical requirements can constrain data sharing, especially in retrospective research. Frameworks such as the European Reference Networks are actively addressing these challenges through federated data approaches and harmonized governance structures, offering promising models for cross-border collaboration.

There is a clear need to increase the proportion of computable data in EHRs to facilitate its reuse for research, ultimately enhancing the feasibility of pediatric and rare disease studies. It may also be valuable to compare the use of EHRs in pediatric care to their use in adult populations. This could help assess whether pediatric data is being underserved compared to adult data, which may hinder its potential for research and clinical trial design.

### Strengths and Limitations

This small-scale, proof-of-concept study aimed to investigate whether routinely collected pediatric health care data are sufficiently rich and structured to support secondary use in research, such as for observational studies, real-world comparator arms, and safety monitoring. A sound methodology was applied to develop an inventory of relevant data items, and a survey was distributed across multiple European clinical sites through the c4c network.

Although the survey was distributed to 21 countries, only 11 (46%) provided feedback, resulting in responses from just 24 sites. Additionally, although the survey was distributed to approximately 270 eligible hospital sites, only 24 responses were received, reflecting a low participation rate. This low participation rate introduces potential selection biases and limits the representativeness and generalizability of the findings. Additionally, there were no formal inclusion or exclusion criteria for site participation. Site selection was based on local discretion and willingness to engage.

Another limitation concerns the development of the data element inventory. While items were reviewed by clinical experts and patient representativeness, no formal prioritization method was used. Although reviewer agreement was high, a structured approach could have improved the focus and feasibility of the final data set. Future studies should consider normal prioritization to enhance methodological rigor.

The survey was designed to be completed at a site level. This allowed multistakeholder input from both clinical and technical personnel. However, because of this design individual-level data (eg, specific job titles or roles of respondents) could not be captured, limiting our ability to assess how respondent background may have influenced the type or accuracy of data reported.

Finally, this study did not evaluate the availability of patient-reported outcome measures, which are important for understanding treatment impact but are rarely integrated into routine EHR systems. Despite these limitations, this study demonstrates the feasibility of engaging a pan-European pediatric research network to assess real-world data availability. The collaborative development and deployment of the survey tool, coupled with strong stakeholder engagement, provide a valuable foundation for future large-scale efforts to improve data standardization and reuse in pediatric research.

### Conclusion and Recommendations

We reported on a pragmatic investigation into whether health care provider organizations (primarily hospitals) that are generally active in undertaking clinical research routinely collect data items on their pediatric patients that would be of high value for designing and conducting clinical trials. This investigation focused on 2 study types that are important in pediatrics, especially in rare diseases: the use of EHR data to establish a real-world comparator arm in a clinical trial and as historical background clinical information for safety studies. Two disease areas, NF and AD, were selected as examples to study.

A total of 24 from 11 countries across Europe and the c4c network completed the survey. We found that a high proportion of the clinical data were routinely collected (apart from lifestyle data); however, much of the relevant information was in free-text entries such as clinic letters, investigation reports, and discharge summaries.

Given the limited scale of this investigation, we recommend that this method be replicated across more pediatric disease areas and with larger hospital site sample sizes to generate more robust evidence of data availability. Natural language processing technologies are needed in the future to extract coded concepts from narratives to have a sufficient level of completeness of data for clinical research purposes.

This investigation could be considered an early call to health care providers, health care professionals, and developers of EHR systems to encourage greater documentation of structured and coded entries to scale up the value of the data for research. It is also likely that better quality structured and coded data could be reused by each health care organization and by health systems for wider quality and safety purposes. However, it is important to find strategies for improving the proportion and quality of structured and coded EHR entries without increasing the data entry burden on clinicians.

## Appendix

Multimedia Appendix 1 Raw data.

Multimedia Appendix 2 Breakdown of additional information.

## Abbreviations

AD: atopic dermatitis
c4c: conect4children
EHDS: European Health Data Space
EHR: electronic health record
EU-PEARL: European Union Patient-Centric Clinical Trial Platforms
GDPR: General Data Protection Regulation
IMI2: Innovative Medicines Initiative
NF: neurofibromatosis
NH: National Hub
RWD: real-world data
SPoC: Single Point of Contact

## References

[1] Giannuzzi V, Stoyanova-Beninska V, Hivert V (2022). Editorial: The use of real world data for regulatory purposes in the rare diseases setting. Front Pharmacol.

[2] Bruneau B, Surdam K, Bland A, Krueger A, Wise A, Cotarlan A, Leviton A, Jouravleva E, Fitzgerald G, Frost HN, Cutler HF, Buddle J, Diaz LG, Cohen M, Sacco NA, Washington R, Mauermann S, Chen V, Bastek A (2024). Redefining feasibility in clinical trials: Collaborative approaches for improved site selection. Contemp Clin Trials Commun.

[3] Rees CA, Pica N, Monuteaux MC, Bourgeois FT (2019). Noncompletion and nonpublication of trials studying rare diseases: A cross-sectional analysis. PLoS Med.

[4] Joshi S (2023). Common Clinical Trial Amendments, why they are submitted and how they can be avoided: a mixed methods study on NHS UK Sponsored Research (Amendments Assemble). Trials.

[5] Macdonald G (2019). Rethinking trials: the pros and cons of protocol amendments. PMLiVE.

[6] De Moor G, Sundgren M, Kalra D, Schmidt A, Dugas M, Claerhout B, Karakoyun T, Ohmann C, Lastic P, Ammour N, Kush R, Dupont D, Cuggia M, Daniel C, Thienpont G, Coorevits P (2015). Using electronic health records for clinical research: the case of the EHR4CR project. J Biomed Inform.

[7] Laaksonen N, Varjonen J, Blomster M, Palomäki A, Vasankari T, Airaksinen J, Huupponen R, Scheinin M, Juuso Blomster (2021). Assessing an Electronic Health Record research platform for identification of clinical trial participants. Contemp Clin Trials Commun.

[8] Palchuk M, London JW, Perez-Rey D, Drebert ZJ, Winer-Jones JP, Thompson CN, Esposito J, Claerhout B (2023). A global federated real-world data and analytics platform for research. JAMIA Open.

[9] Dhaenens B, Heimann G, Bakker A, Nievo M, Ferner R, Evans DG, Wolkenstein P, Leubner J, Potratz C, Carton C, Iloeje U, Kirk G, Blakeley JO, Plotkin S, Fisher MJ, Kim A, Driever PH, Azizi AA, Widemann BrC, Gross A, Parke T, Legius E, Oostenbrink R (2024). Platform trial design for neurofibromatosis type 1, NF2-related schwannomatosis and non-NF2-related schwannomatosis: A potential model for rare diseases. Neurooncol Pract.

[10] Gross AM, Dombi E, Fisher PE, Song Y, Landsman BD, Listernick ER, Fisher M, Plotkin SR, Cai KD, Blakeley JO, Ostrow A-MK, Widemann BC (2020). Selumetinib in children with inoperable plexiform neurofibromas. N Engl J Med.

[11] Gross AM (2021). Curr Probl Cancer.

[12] Velummailum RR, McKibbon C, Brenner DR, Stringer EA, Ekstrom L, Dron L (2023). Data challenges for externally controlled trials: Viewpoint. J Med Internet Res.

[13] Doods J, Botteri F, Dugas M, Fritz F, EHR4CR WP7 (2014). A European inventory of common electronic health record data elements for clinical trial feasibility. Trials.

[14] Cash-Gibson L, Pericas JM, Spiertz C, van de Ketterij E, Molero E, Patalano F, Kalra D, Ussi A, Van Dessel A, Genescà J (2021). EU-PEARL: Changing the paradigm of clinical trials in Europe. Eur J Public Health.

[15] Lombardo G, Couvert C, Kose M, Begum A, Spiertz C, Worrell C, Hasselbaink D, Didden E, Sforzini L, Todorovic M, Lewi M, Brown M, Vaterkowski M, Gullet N, Amasi-Hartoonian N, Griffon N, Pais R, Rodriguez Navarro S, Kremer A, Maes C, Tan EH, Moinat M, Ferrer JG, Pariante CM, Kalra D, Ammour N, Kalko S (2023). Electronic health records (EHRs) in clinical research and platform trials: Application of the innovative EHR-based methods developed by EU-PEARL. J Biomed Inform.

[16] Hedley V, Leary R, Sen A, Irvin A, Heslop E, Straub V, Gasthuys E, Allegaert K, Dossche L, Turner M (2024). Performing clinical drug trials in children with a rare disease. Essentials of Translational Pediatric Drug Development.

[17] (2024). Joint HMA/EMA Big Data Steering Group workshop on real-world evidence (RWE) methods. European Medicines Agency.

[18] Sen A, Hedley V, Owen J, Cornet R, Kalra D, Engel C, Palmeri A, Lee J, Roze J, Standing J, Warris A, Pansieri C, Leary R, Turner M, Straub V (2023). Standardizing paediatric clinical data: The development of the conect4children (c4c) cross cutting paediatric data dictionary. J Soc Clin Data Manag.

[19] Owen J, Sen A, Aurich B, Engel C, Cavallaro G, Degraeuwe E, Kalra D, Cornet R, Walsh M, Berkery T, Palmeri A, Mahler F, Malik S, Persijn L, Amadi C, Thuet J, Woodworth S, Nally S, Leary R, Marshall R, Straub V (2024). Development of the CDISC Pediatrics User Guide: a CDISC and conect4children collaboration. Front Med (Lausanne).

[20] Palmeri A, Leary R, Sen A, Cornet R, Welter D, Rocca-Serra P (2023). Creating a metadata profile for clinical trial protocols. FAIR Cookbook.

[21] Sen A, Hedley V, Degraeuwe E, Hirschfeld S, Cornet R, Walls R, Owen J, Robinson PN, Neilan EG, Liener T, Nisato G, Modi N, Woodworth S, Palmeri A, Gaentzsch R, Walsh M, Berkery T, Lee J, Persijn L, Baker K, An Haack K, Segovia Simon S, Jacobsen JOB, Reggiardo G, Kirwin MA, Trueman J, Pansieri C, Bonifazi D, Nally S, Bonifazi F, Leary R, Straub V (2024). Learning from conect4children: A collaborative approach towards standardisation of disease-specific paediatric research data. Data.

[22] Turner MA, Kalra D, Cornet R, Palmeri A, Sen A, Owen J, Pansieri C, Lee J, Hedley V, Nally S, Bonifazi F, Leary R, Straub V (2025). Paediatric-specific content in data standards for health. Arch Dis Child.

[23] Turner MA, Hildebrand H, Fernandes RM, de Wildt SN, Mahler F, Hankard R, Leary R, Bonifazi F, Nobels P, Cheng K, Attar S, Rossi P, Rocchi F, Claverol J, Nafria B, Giaquinto C (2021). The conect4children (c4c) consortium: Potential for improving European clinical research into medicines for children. Pharmaceut Med.

[24] Bonacin R, de Figueiredo EB, de Franco Rosa F, Dos Reis JC, Dametto M (2024). The reuse of electronic health records information models in the oncology domain: Studies with the bioframe framework. J Biomed Inform.

[25] Post AR, Burningham Z, Halwani AS (2022). Electronic health record data in cancer learning health systems: challenges and opportunities. JCO Clin Cancer Inform.

[26] Nordo AH, Levaux HP, Becnel LB, Galvez J, Rao P, Stem K, Prakash E, Kush RD (2019). Use of EHRs data for clinical research: Historical progress and current applications. Learn Health Syst.

