# Supplementary material for: The Potential to Leverage Real-World Data for Pediatric Clinical Trials: A Proof-of-Concept Study
Source: J Med Internet Res. 2025 May 30;27:e72573. doi: 10.2196/72573 (PMC12166320; doi:10.2196/72573)
Supplement: Multimedia Appendix 1 [file jmir_v27i1e72573_app1.docx]

**Multimedia Appendix #1**

| Demographics and diagnose | | | | | | | |
| --- | --- | --- | --- | --- | --- | --- | --- |
|  | **No answer** | **Not collected** | **Derived from other EHR data** | **Paper charts** | **Separate system not linked to EHR** | **Free text** | **Structured and coded** |
| Date of diagnosis | / | 4,17 % | / | / | / | 50 % | 45,83 % |
| Gestational age of birth | 4,18 % | 8,33 % | / | / | / | 37,5 % | 50 % |
| Pediatric diagnosis | / | 4,17 % | / | / | / | 37,5 % | 58,33 % |
| Birth weight | 4,18 % | 8,33 % | / | / | / | 16,66 % | 70,83 % |
| Sex | / | / | / | / | / | 4,17 % | 95,83 % |
| Date of birth | / | / | / | / | / | 4,17 % | 95,83 % |
|  | Neurofibromatosis | | | | | | |
|  | **No answer** | **Not collected** | **Derived from other EHR data** | **Paper charts** | **Separate system not linked to EHR** | **Free text** | **Structured and coded** |
| Genetically pathogen variant | 45,83 % | 8,33 % | 4,17 % | 4,17 % | / | 33,33 % | 4,17 % |
| Sphenoid dysplasia | 37,5 % | 8,33 % | 4,17 % | / | / | 41,67 % | 8,33 % |
| Pseuodoarthrosis of a long bone | 37,5 % | 8,33 % | 4,17 % | / | / | 41,67 % | 8,33 % |
| Paraspinal plexiform neurofibroma | 37,5 % | 8,33 % | 4,17 % | / | / | 41,67 % | 8,33 % |
| Optic pathway glioma | 37,5 % | 8,33 % | 4,17 % | / | / | 41,67 % | 8,33 % |
| Distinctive issues leasions | 37,5 % | 8,33 % | 4,17 % | / | / | 41,67 % | 8,33 % |
| Anterolateral bowing of the tibia | 37,5 % | 8,33 % | 4,17 % | / | / | 41,67 % | 8,33 % |
| Plexifirm neurofibroma | 37,5 % | 4,17 % | 4,16 % | / | / | 41,67 % | 12,5 % |
| NF1 diagnosis genetically confirmed | 37,5 % | 12,5 % | / | 4,17 % | / | 33,33 % | 12,5 % |
| Low grade glioma | 37,5 % | 4,17 % | 4,16 % | / | / | 41,67 % | 12,5 % |
| High grade glioma | 37,5 % | 4,17 % | 4,16 % | / | / | 41,67 % | 12,5 % |
| Cutaneous neurofibroma | 37,5 % | 4,17 % | 4,16 % | / | / | 41,67 % | 12,5 % |
|  | Atopic dermatitis | | | | | | |
|  | **No answer** | **Not collected** | **Derived from other EHR data** | **Paper charts** | **Separate system not linked to EHR** | **Free text** | **Structured and coded** |
| Presence of burning | 29,17 % | 4,17 % | 8,33 % | / | / | 58,33 % | / |
| Presence of blisters | 29,17 % | 8,33 % | / | / | / | 62,5 % | / |
| Presence of stinging | 29,17 % | 4,16 % | 4,16 % | / | / | 58,33 % | 4,17 % |
| Presence of skin irritation | 29,17 % | / | 8,33 % | / | / | 58,33 % | 4,17 % |
| Presence of non-skin atopic symptoms | 29,17 % | / | 8,33 % | / | / | 58,33 % | 4,17 % |
| Presence of nocturnal scratching | 29,17 % | / | 8,33 % | / | / | 58,33 % | 4,17 % |
| Presence of exacerbation of pruritus | 29,17 % | / | 8,33 % | / | / | 58,33 % | 4,17 % |
| Presence of exacerbation of eczema | 29,17 % | / | 8,33 % | / | / | 58,33 % | 4,17 % |
| Presence of erythematous rash | 29,17 % | / | 8,33 % | / | / | 58,33 % | 4,17 % |
| Presence of dusky erythema | 29,17 % | / | 8,33 % | / | / | 58,33 % | 4,17 % |
| Presence of urticaria | 29,17 % | / | 4,17 % | / | / | 58,33 % | 8,33 % |
| Presence of infected eczema | 29,17 % | / | 4,17 % | / | / | 58,33 % | 8,33 % |
| Presence of folliculitits | 29,17 % | 4,17 % | / | / | / | 58,33 % | 8,33 % |
| Presence of herpes simplex | 29,17 % | 4,17 % | / | / | / | 54,17 % | 12,5 % |
|  | Drug and vaccine safety | | | | | | |
|  | **No answer** | **Not collected** | **Derived from other EHR data** | **Paper charts** | **Separate system not linked to EHR** | **Free text** | **Structured and coded** |
| Outcome of error | 16,67 % | 4,17 % | 8,34 % | 8,34 % | / | 58,33 % | 4,17 % |
| Laboratory test results | 20,83 % | 4,17 % | 4,17 % | 8,34 % | / | 58,33 % | 4,17 % |
| Date of error | 20,83 % | 4,17 % | 4,17 % | 8,34 % | 4,17 % | 54,17 % | 4,17 % |
| Clinical sign or abnormal test results due to medication error | 12,5 % | 4,17 % | 8,34 % | 4,17 % | 8,34 % | 58,33 % % | 4,17 % |
| Treatment administered for reaction | 12,5 % | 4,17 % | 8,34 % | 4,17 % | / | 62,5 % | 8,33 % |
| Reaction confirmed by health care provider | 12,5 % | / | 8,34 % | 4,17 % | / | 66,67 % | 8,33 % |
| Medication error | 12,5 % | / | 12,5 % | 4,17 % | / | 62,5 % | 8,33 % |
| Date of resolution, if applicable | 20,83 % | 4,17 % | 4,17 % | 8,34 % | 4,17 % | 50 % | 8,33 % |
| Date of resolution of reaction | 12,5 % | / | 12,5 % | 4,17 % | / | 58,33 % | 12,5 % |
| Date of onset of reaction | 12,5 % | 4,17 % | 8,34 % | 8,34 % | / | 54,17 % | 12,5 % |
| Vaccine brand name | 12,5 % | 12,5 % | 8,34 % | / | / | 50 % | 16,67 % |
| Outcome of reaction | 12,5 % | / | 8,34 % | 4,17 % | / | 58,33 % | 12,67 % |
| Reasons for stopping medication (if applicable) | 12,5 % | 12,5 % | 8,34 % | / | / | 45,83 % | 20,83 % |
| Date of investigation or test | 12,5 % | / | 4,17 % | / | / | 62,5 % | 20,83 % |
| Severity of reaction | 12,5 % | 4,17 % | 4,17 % | 4,17 % | / | 50 % | 25 % |
| Indication of medication | 8,33 % | 4,17 % | 8,34 % | / | / | 45,83 % | 33,33 % |
| How the dose was calculated | 20,83 % | 8,33 % | 4,17 % | 4,17 % | 4,17 % | 20,83 % | 37,5 % |
| Date of vaccination | 12,5 % | 20,83 % | / | / | 4,17 % | 25 % | 37,5 % |
| Results of development assessment | 12,5 % | / | / | 4,17 % | 4,17 % | 20,83 % | 58,33 % |
| Brand name of medication | 12,5 % | 16,67 % | 4,16 % | / | 4,16 % | 16,67 % | 45,83 % |
| Lot number of vaccine | 12,5 % | 16,67 % | / | 4,16 % | / | 16,67 % | 50 % |
| Adverse drug or vaccine reactions | 12,5 % | 4,17 % | / | 4,17 % | / | 20,83 % | 58,33 % |
| Stop date | 12,5 % | / | 4,17 % | 4,17 % | / | 20,83 % | 58,33 % |
| Start date | 12,5 % | / | 4,17 % | 4,17 % | / | 20,83 % | 58,33 % |
| Route of administration | 12,5 % | / | 4,17 % | 4,17 % | / | 20,83 % | 58,33 % |
| Imaging results | 12,5 % | / | / | 4,17 % | 4,17 % | 20,83 % | 58,33 % |
| Generic name of medication | 12,5 % | 4,17 % | / | 4,17 % | / | 20,83 % | 58,33 % |
| Frequency of administration | 12,5 % | / | 4,17 % | 4,17 % | / | 20,83 % | 58,33 % |
| Dose of medication | 12,5 % | / | 4,17 % | 4,17 % | / | 20,83 % | 58,33 % |
| Vital signs | 16,67 % | / | / | / | 4,16 % | 12,5 % | 66,67 % |
| Relevant family conditions | | | | | | | |
|  | **No answer** | **Not collected** | **Derived from other EHR data** | **Paper charts** | **Separate system not linked to EHR** | **Free text** | **Structured and coded** |
| History of substance abuse problems in the mother | 12,5 % | 20,83 % | 4,17 % | / | / | 62,5 % | / |
| History of substance abuse problems in the father | 12,5 % | 20,83 % | 4,17 % | / | / | 62,5 % | / |
| History of learning difficulty in the mother | 12,5 % | 33,33 % | 4,17 % | / | / | 50 % | / |
| History of learning difficulty in the father | 12,5 % | 33,33 % | 4,17 % | / | / | 50 % | / |
| History of psychiatric disease in the mother | 12,5 % | 16,67 % | 4,17 % | / | / | 62,49 % | 4,17 % |
| History of psychiatric disease in the father | 12,5 % | 16,67 % | 4,17 % | / | / | 62,49 % | 4,17 % |
| Relevant past procedure | | | | | | | |
|  | **No answer** | **Not collected** | **Derived from other EHR data** | **Paper charts** | **Separate system not linked to EHR** | **Free text** | **Structured and coded** |
| Procedure outcome | 12,5 % | 8,33 % | 8,33 % | / | / | 41,67 % | 29,17 % |
| Indication for the procedure | 12,5 % | / | 8,33 % | / | / | 50 % | 29,17 % |
| Provider type | 12,5 % | 29,17 % | 8,33 % | / | / | 12,5 % | 37,5 % |
| Procedure(s) type | 12,5 % | / | 4,17 % | / | / | 45,83 % | 37,5 % |
| Encounter type | 12,5 % | 18,75 % | 8,34 % | / | / | 18,74 % | 41,67 % |
| Date of procedure | 12,5 % | / | 4,17 % | / | / | 50 % | 41,67 % |
| Presence other conditions | | | | | | | |
|  | **No answer** | **Not collected** | **Derived from other EHR data** | **Paper charts** | **Separate system not linked to EHR** | **Free text** | **Structured and coded** |
| Pregnancy conditions of the mother | / | 8,33 % | 8,33 % | / | / | 75,01 % | 8,33 % |
| History of substance abuse problems related to the patient | 16,67 % | 12,5 % | / | / | / | 41,68 % | 8,33 % |
| Birth complications of the mother | 4,17 % | 12,5 % | 8,33 % | / | / | 56,67 % | 8,33 % |
| Perinatal events of the mother | 4,17 % | 8,33 % | 8,33 % | / | / | 66,67 % | 12,5 % |
| History of psychiatric disorder related to the patient | 16,67 % | 8,34 % | 4,17 % | / | / | 41,68 % | 12,5 % |
| History of development delay related to the patient | 16,67 % | 8,34 % | 4,17 % | / | / | 41,68 % | 12,5 % |
| Multiple births by the mother | / | 8,33 % | 8,33 % | / | / | 58,37 % | 25 % |
| Lifestyle and social status | | | | | | | |
|  | **No answer** | **Not collected** | **Derived from other EHR data** | **Paper charts** | **Separate system not linked to EHR** | **Free text** | **Structured and coded** |
| Infant exposure to smoking | 4,17 % | 33,33 % | / | / | / | 54,17 % | 8,33 % |
| Personal smoking history | 4,17 % | 25 % | / | / | / | 58,33 % | 12,5 % |
| Care givers education level | 4,17 % | 45,83 % | / | / | / | 37,5 % | 12,5 % |
| Feeding type | 4,17 % | 20,83 % | / | / | / | 58,33 % | 16,67 % |
| Allergies | | | | | | | |
|  | **No answer** | **Not collected** | **Derived from other EHR data** | **Paper charts** | **Separate system not linked to EHR** | **Free text** | **Structured and coded** |
| Infant exposure to food allergens | 16,67 % | 20,83 % | 4,17 % | / | / | 54,16 % | 4,17 % |
| Exposure to aeroallergens/irritants | 16,67 % | 29,16 % | / | / | / | 41,67 % | 12,5 % |
| Conunctivitis (AD specific) | 16,67 % | 4,17 % | 4,17% | 4,17 % | / | 54,15 % | 16,67 % |
| Confirmatory testing | 16,67 % | 8,33 % | 4,17 % | 4,17 % | / | 50 % | 16,67 % |
| Confirmation by a healthcare provider | 16,67 % | 12,5 % | 4,17 % | 4,17 % | / | 4,66 % | 20,83 % |
| Other allergies | 16,67 % | / | 4,17 % | / | / | 54,17 % | 25 % |
| Food allergies | 16,67 % | / | 4,17 % | / | / | 41,66 % | 37,5 % |
| History of hypersensitivity to medications | 16,67 % | / | 4,17 % | / | / | 29,17 % | 50 % |
| Current findings | | | | | | | |
|  | **No answer** | **Not collected** | **Derived from other EHR data** | **Paper charts** | **Separate system not linked to EHR** | **Free text** | **Structured and coded** |
| Bacterial, viral or fungal infections (AD specific) | 4,17 % | 8,34 % | / | 8,34 % | / | 45,84 % | 33,34 % |
| Oxygen saturation | 4,17 % | 8,34 % | / | 4,17 % | / | 24,98 % | 58,34 % |
| Respiratory rate |  |  |  |  |  |  |  |
| Temperature | 4,17 % | 4,17 % | / | 4,17 % | / | 20,82 % | 66, 67 % |
| Pulse rate | 4,17 % | 4,17 % | / | 4,17 % | / | 20,82 % | 66, 67 % |
| Heart rate | 4,17 % | 8,34 % | / | 4,17 % | / | 16,65 % | 66, 67 % |
| Head circumference | 4,17 % | 4,17 % | 4,17 % | 4,17 % | / | 16,65 % | 66, 67 % |
| Height (or length for babies) | 4,17 % | 4,17 % | 4,17 % | / | / | 18,82 % | 68,67 % |
| The data of these measurements | 4,17 % | 4,17 % | 4,17 % | / | / | 18,82 % | 68,67 % |
| The data of these measurements | 4,17 % | 4,17 % | 4,17 % | / | / | 18,82 % | 68,67 % |
| Systolic blood pressure | 4,17 % | 4,17 % | 4,17 % | / | / | 18,82 % | 68,67 % |
| Diastolic blood pressure | 4,17 % | 4,17 % | 4,17 % | / | / | 18,82 % | 68,67 % |
| Weight | 4,17 % | / | / | 4,17 % | / | 12,49 % | 79,17 % |
| Encounter history | | | | | | | |
|  | **No answer** | **Not collected** | **Derived from other EHR data** | **Paper charts** | **Separate system not linked to EHR** | **Free text** | **Structured and coded** |
| Type of transport | 16,67 % | 20,84 | / | / | / | 24,99 % | 37,5 % |
| Encounter type: study observation | 16,67 % | 8,34 % | / | 4,17 % | / | 22,87 % | 47,95 % |
| Source of referral | 16,67 % | / | / | / | / | 33,33 % | 50 % |
| Encounter type: primary/community care | 16,67 % | 20,83 % | / | / | / | 12,5 % | 50 % |
| Provider/facility type | 16,67 % | / | / | 4,17 % | / | 16,67 % | 62,49 % |
| Location of transfer | 16,67 % | 4,17 % | / | / | / | 16,67 % | 62,49 % |
| If currently participating in clinical trial | 12,5 % | 4,17 % | / | / | / | 20,84 % | 62,49 % |
| Date of transfer | 16,67 % | 4,17 % | / | / | / | 16,67 % | 62,49 % |
| Date of discharge | 16,67 % | 16,67 % | / | / | / | / | 66,66 % |
| Encounter type: hospital admission | 16,67 % | / | / | / | / | 12,5 % | 70,83 % |
| Encounter type: ambulatory clinic | 16,67 % | / | / | / | / | 12,5 % | 70,83 % |
| Date of encounter or observation | 16,67 % | / | / | / | / | 12,5 % | 70,83 % |
| Date of discharge | 16,67 % | / | / | / | / | 12,5 % | 70,83 % |
| Date of admission | 16,67 % | / | / | / | / | 12,5 % | 70,83 % |
| If PN is present | | | | | | | |
|  | **No answer** | **Not collected** | **Derived from other EHR data** | **Paper charts** | **Separate system not linked to EHR** | **Free text** | **Structured and coded** |
| Type of plexiform neurofibroma complications | 41,67 % | 4,17 % | 4,17 % | / | / | 45,82 % | 4,17 % |
| Tumour size on MRI: longest diameter | 41,67 % | 4,17 % | 8,34 % | / | 4,17 % | 37,48 % | 4,17 % |
| MRI scan results | 37,5 % | 4,17 % | 4,17 % | / | 4,17 % | 45,83 % | 4,17 % |
| Therapy received for plexiform neurofibroma | 41,67 % | 4,17 % | 4,17 % | / | / | 41,65 % | 8,34 % |
| Location of plexiform neurofibroma | 41,67 % | 4,17 % | 4,17 % | / | / | 41,65 % | 8,34 % |
| If LGG is present | | | | | | | |
|  | **No answer** | **Not collected** | **Derived from other EHR data** | **Paper charts** | **Separate system not linked to EHR** | **Free text** | **Structured and coded** |
| RAPNO criteria | 41,66 % | 4,17 % | 4,17 % | / | 8,34 % | 41,66 % | / |
| Tumour type | 41,66 % | 4,17 % | / | 4,17 % | / | 37,5 % | 12,5 % |
| Tumour location | 41,66 % | 4,17 % | 4,17 % | / | / | 41,66 % | 8,34 % |
| MRI scan results | 37,49 % | 4,17 % | 4,17 % | / | 4,17 % | 45,83 % | 4,17 % |
| Therapy received | 41,66 % | 4,17 % | 4,17 % | / | / | 41,66 % | 8,34 % |
| Diagnosis | 41,66 % | 4,17 % | / | 4,17 % | / | 37,5 % | 12,5 % |
| If OPG is present | | | | | | | |
|  | **No answer** | **Not collected** | **Derived from other EHR data** | **Paper charts** | **Separate system not linked to EHR** | **Free text** | **Structured and coded** |
| RAPNO criteria | 41,67 % | 4,17 % | 8,34 % | / | 8,34 % | 37,48 % | / |
| Dodge criteria | 41,67 % | 12,5 % | 8,34 % | / | 8,34 % | 29,15 % | / |
| MRI scan results | 37,5 % | / | 4,17 % | / | 4,17 % | 45,82 % | 8,34 % |
| Relevant family conditions related to Neurofibromatosis | | | | | | | |
|  | **No answer** | **Not collected** | **Derived from other EHR data** | **Paper charts** | **Separate system not linked to EHR** | **Free text** | **Structured and coded** |
| Neurofibromatosis related to the siblings | 41,64 % | / | 4,18 % | / | / | 54,18 % | / |
| Neurofibromatosis related to the mother | 41,64 % | / | 4,18 % | / | / | 54,18 % | / |
| Neurofibromatosis related to the father | 41,64 % | / | 4,18 % | / | / | 54,18 % | / |
| Relevant family conditions related to Atopic Dermatitis | | | | | | | |
|  | **No answer** | **Not collected** | **Derived from other EHR data** | **Paper charts** | **Separate system not linked to EHR** | **Free text** | **Structured and coded** |
| Atopic dermatitis related to the mother | 25 % | 4,18 % | 4,18 % | / | / | 50 % | 4,18 % |
| Atopic dermatitis related to the father | 25 % | 4,18 % | 4,18 % | / | / | 50 % | 4,18 % |
| Atopic dermatitis related to siblings | 25 % | 4,18 % | 4,18 % | / | / | 50 % | 4,18 % |
| Phototherapy – Atopic dermatitis specific | | | | | | | |
|  | **No answer** | **Not collected** | **Derived from other EHR data** | **Paper charts** | **Separate system not linked to EHR** | **Free text** | **Structured and coded** |
| Side effect | 41,67 % | 4,17 % | / | / | / | 45,82 % | 8,34 % |
| Outcome | 41,67 % | 4,17 % | / | / | / | 45,82 % | 8,34 % |
| Target area | 41,67 % | 8,34 % | / | / | / | 37,49 % | 12,5 % |
| Stop date | 41,67 % | 4,17 % | / | / | / | 41,66 % | 12,5 % |
| Start date | 41,67 % | 4,17 % | / | / | / | 41,66 % | 12,5 % |
| Indication | 41,67 % | 4,17 % | / | / | / | 41,66 % | 12,5 % |
| Frequency | 41,67 % | 8,34 % | / | / | / | 33,32 % | 16,67 % |
| Dose calculation method | 41,67 % | 12,5 % | / | / | / | 29,16 % | 16,67 % |
| Dose | 41,67 % | 4,17 % | / | / | / | 37,49 % | 16,67 % |
| Radiotherapy – Neurofibromatosis specific | | | | | | | |
|  | **No answer** | **Not collected** | **Derived from other EHR data** | **Paper charts** | **Separate system not linked to EHR** | **Free text** | **Structured and coded** |
| Side effects | 41,67 % | / | 4,17 % | / | / | 50 % | 4,17 % |
| Target volume | 41,67 % | 4,17 % | 4,17 % | 4,17 % | / | 37,48 % | 8,34 % |
| Stop date | 41,67 % | / | 4,17 % | / | / | 41,66 % | 8,34 % |
| Outcome | 41,67 % | / | 4,17 % | / | / | 41,66 % | 8,34 % |
| Indication | 41,67 % | / | 4,17 % | / | / | 41,66 % | 8,34 % |
| Start date | 41,67 % | / | 4,17 % | / | / | 41,66 % | 12,5 % |
| Route of administration | 41,67 % | / | 4,17 % | 4,17 % | / | 37,49 % | 12,5 % |
| Frequency | 41,67 % | / | 4,17 % | 4,17 % | / | 37,49 % | 12,5 % |
| Dose calculation method | 41,67 % | 4,17 % | 4,17 % | 4,17 % | / | 33,32 % | 12,5 % |
| Dose | 41,67 % | / | 4,17 % | / | / | 41,67 % | 12,5 % |
