# Supplementary material for: The Potential to Leverage Real-World Data for Pediatric Clinical Trials: A Proof-of-Concept Study
Source: J Med Internet Res. 2025 May 30;27:e72573. doi: 10.2196/72573 (PMC12166320; doi:10.2196/72573)
Supplement: Multimedia Appendix 2 [file jmir_v27i1e72573_app2.docx]

**Multimedia Appendix #2**

*Demographics and Diagnosis*

Data items related to demographics and diagnosis showed high consistency in structured and coded formats for essential variables like date of birth (95.83 %) and sex (95.83 %). Birth weight, paediatric diagnosis, and gestational age were less consistently collected in a structured format, with some being derived from other EHR data or recorded as free text. For paediatric diagnoses, 37.5% were recorded as free text, while only 58.33% were structured. This emphasized variability in how general patient demographic data is managed across sites, which may affect the standardization and accessibility of critical patient information. Annex 1 provides an in-depth view of these results, and figure 1 presents the summarized data for this section.

Participants were asked questions about the presence of data items within their EHR system. Some of these data items specifically related to the case studies we were exploring AD, NF and Post-Marketing Surveillance. Within the results section of this report, some sections include results for all case studies, which have been grouped and analysed together. These sections are relevant family conditions, relevant past procedures, presence of other conditions, lifestyle and social status, allergies, current findings and encounter history.

Figure 1: Percentage-stacked bar plot for Demographics and Diagnosis data items. Each colour represents the percentage of sites collecting the data items in a particular format.


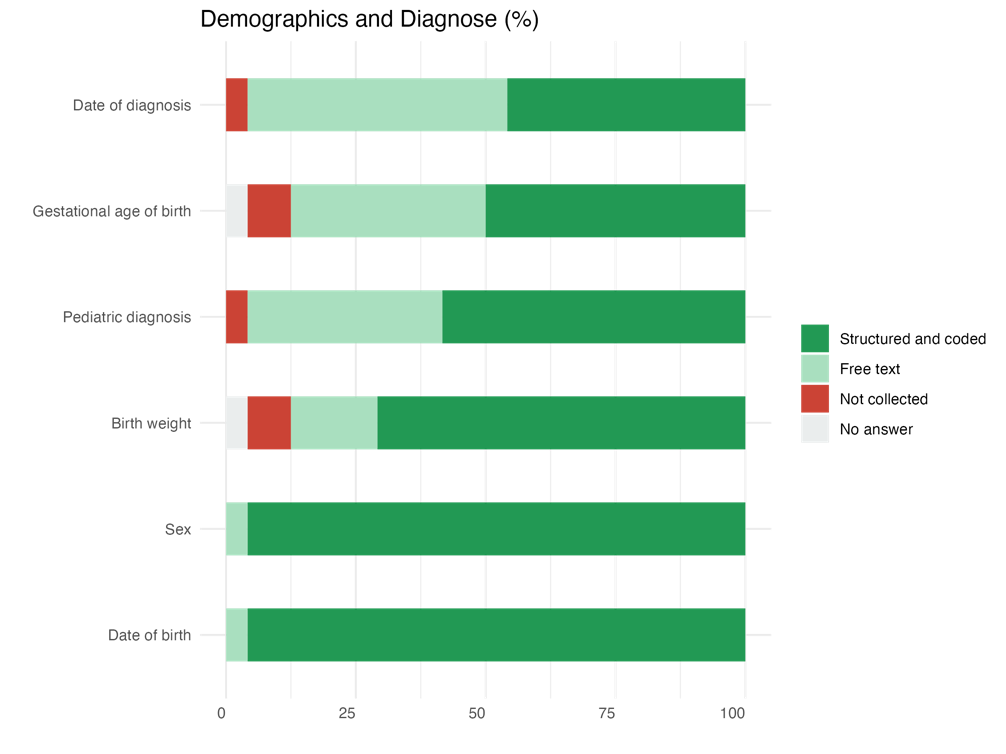
*Low Grade Glioma (LGG) data*

For LGG, approximately 41.66% of sites collected structured data on diagnosis and therapy, but imaging (MRI) results and tumour types were mostly recorded as free text. With only 12.5% of tumour type data structured, there were significant gaps in how low-grade glioma was tracked, which may impact research and care standardization. Further insights are detailed in Annex 1, and the summarized results can be seen in figure 2.


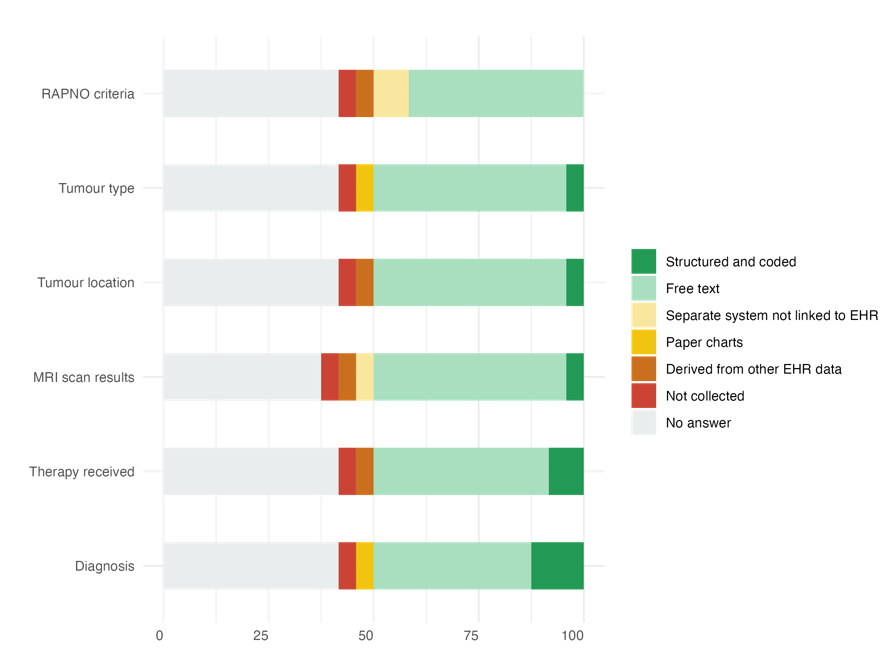


Figure 2: Percentage-stacked bar plot for Low Grade Glioma (LGG) data items. Each colour represents the percentage of sites collecting the data items in a particular format.

*Optic Pathway Glioma (OPG) data*

Sites reported MRI scan results for OPG in structured formats at 45.82%, but there were gaps in the use of RAPNO or Dodge criteria, with only 37.48% and 29.15%, respectively, using structured formats. The lack of systematic data capture in key diagnostic criteria may hinder cross-comparison of treatment outcomes. Annex 1 provides more in-depth information, and figure 3 summarizes these findings.


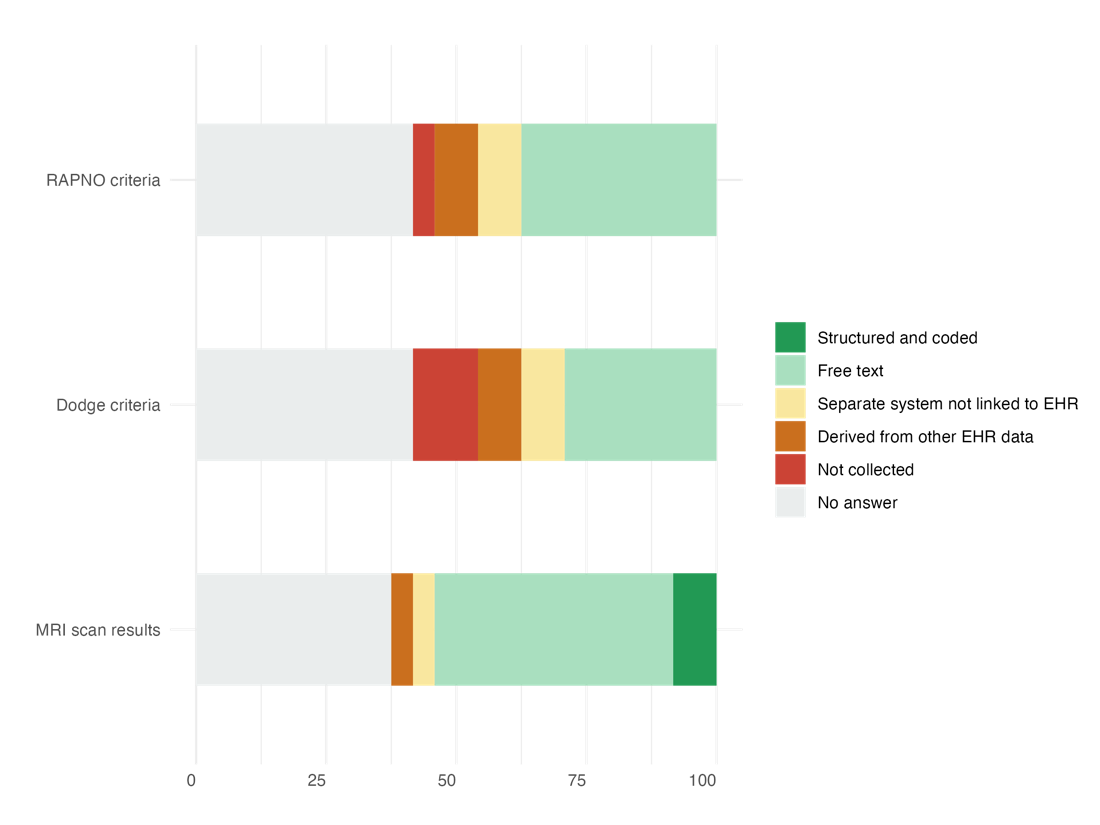


Figure 3: Percentage-stacked bar plot for Optic Pathway Glioma (OPP) data items. Each colour represents the percentage of sites collecting the data items in a particular format.

*Plexiform Neurofibroma (PN) data*

For PN data, approximately 41.67% of sites captured information like location, therapy, and MRI scan results in free text, with only 8.34% structured. A considerable portion of data was not collected at all, making the systematic tracking of PN difficult across sites. The reliance on free-text data further complicates longitudinal patient management and outcome assessment. A more detailed analysis is available in Annex 1, and figure 4 summarizes these results.


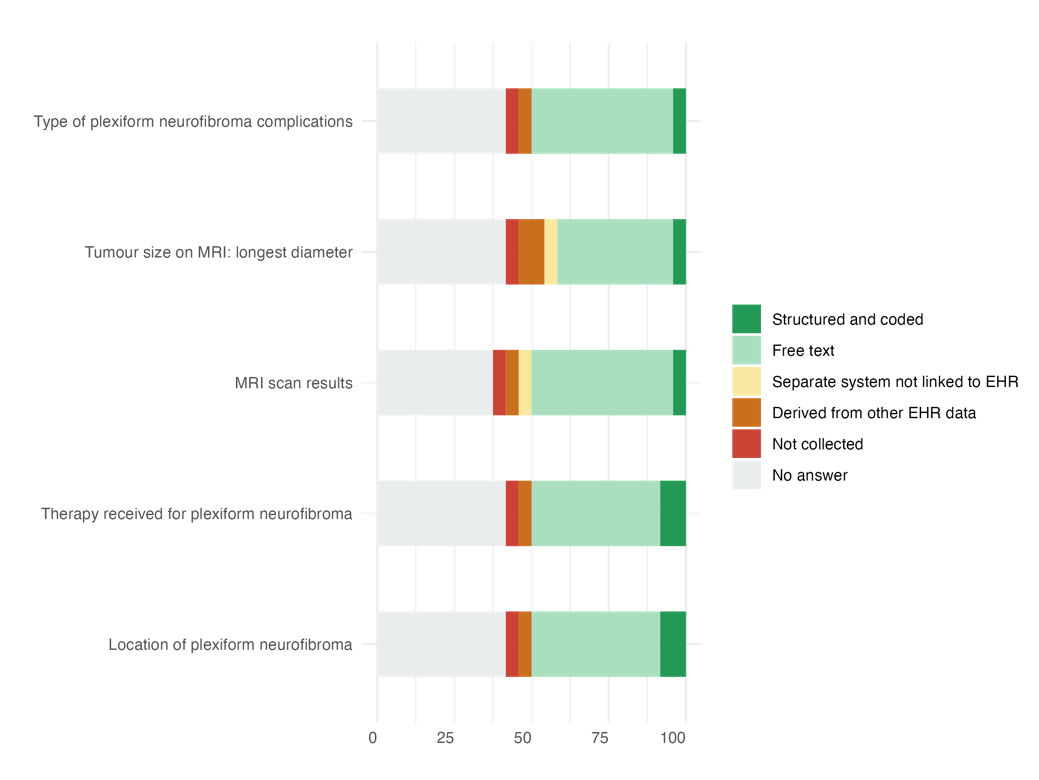


Figure 4: Percentage-stacked bar plot for Plexiform Neurofibroma (PN) data items. Each colour represents the percentage of sites collecting the data items in a particular format.

*Relevant family conditions*

Family history variables, including psychiatric disease and substance abuse, were primarily collected in structured formats, but a notable portion of data was not collected or was derived from other EHR sources. For instance, psychiatric history of the father and mother was structured in 62.49% of responses, but a substantial number of sites reported not collecting this information. This variability may affect the understanding of hereditary or environmental factors influencing disease progression in both NF and AD patients. Annex 1 contains more detailed results, and figure 5 presents a summary of the findings.


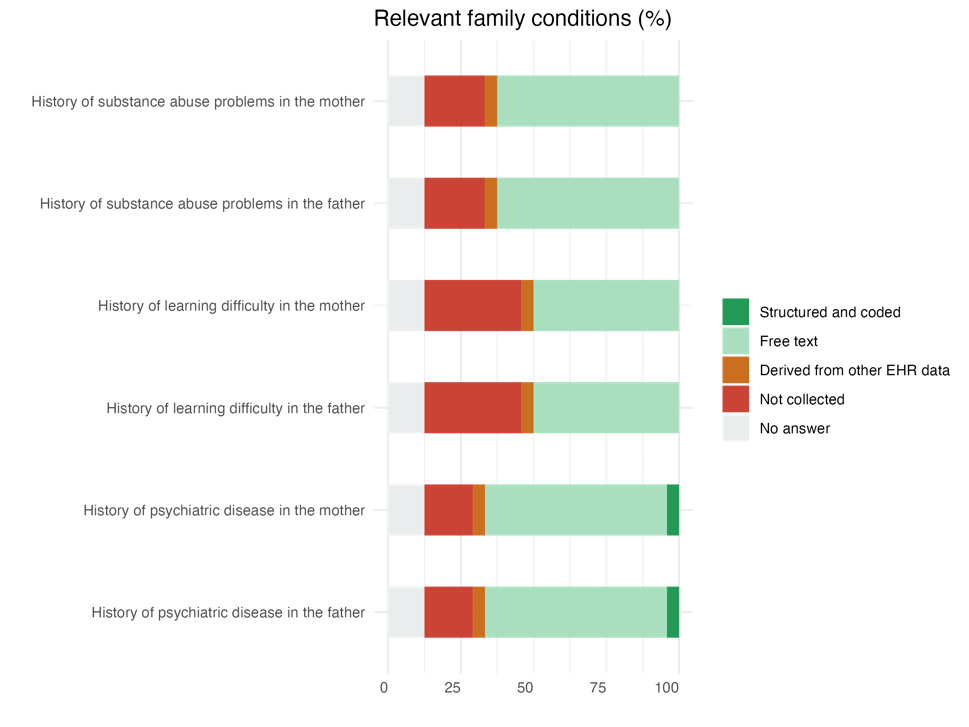


Figure 5: Percentage-stacked bar plot for relevant family conditions data items. Each colour represents the percentage of sites collecting the data items in a particular format.

*Family conditions in Atopic dermatitis (AD)*

Data related to family history for AD showed that 50% of sites captured information about family members’ history of AD in a structured format. A smaller proportion of sites relied on derived or free-text data, highlighting variability in documenting familial links to the disease. Annex 1 contains a more detailed view, and figure 6 provides a visual summary.


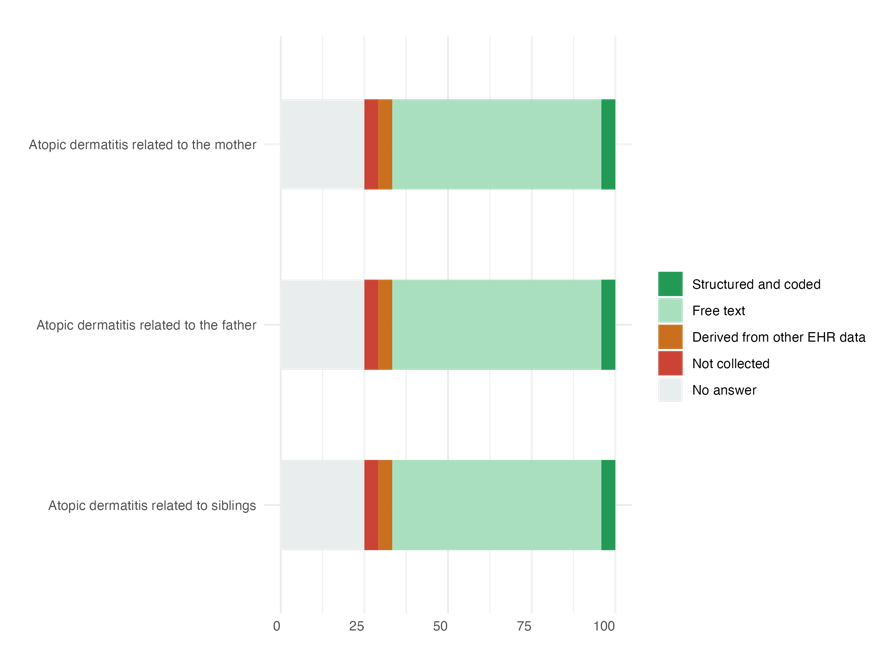


Figure 6: Percentage-stacked bar plot for family conditions in Atopic Dermatitis (AP) data items. Each colour represents the percentage of sites collecting the data items in a particular format.

*Family conditions in Neurofibromatosis (NF)*

For family history data related to NF, approximately 54.18% of sites collected structured data, but 41.64% left these items unanswered. This suggests significant data gaps, which may limit the ability to understand the hereditary nature of the condition across families. Further details are available in Annex 1, with figure 7 summarizing the results.


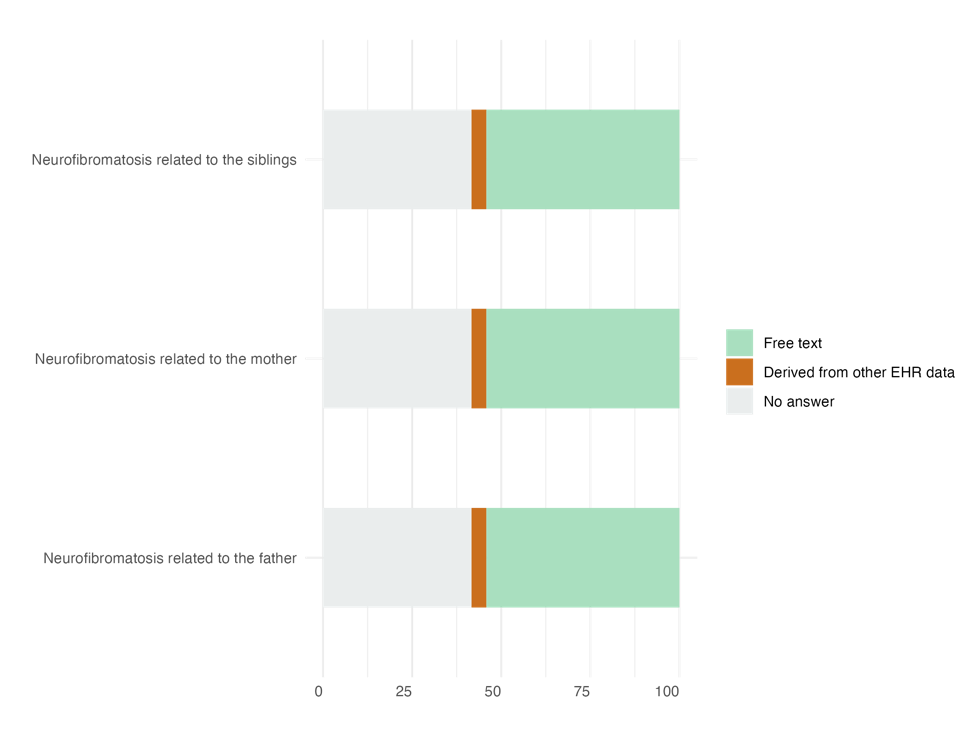


Figure 7: Percentage-stacked bar plot for family conditions in Neurofibromatosis (NF) data items. Each colour represents the percentage of sites collecting data items in a particular format.

*Relevant past procedures*

Data items on procedures, including the date and type of procedures performed, were predominantly collected in structured formats, though some sites left questions unanswered. Around 50% of sites captured structured procedure data, with additional entries left blank or collected on paper charts. Indications for procedures and outcomes were also largely structured (50% and 41.67%, respectively). However, gaps in reporting on the provider type and procedure indication suggest inconsistencies in documentation, which may impact procedural outcome analysis. For a more detailed view, see Annex 1 and refer to figure 8 for a summary.


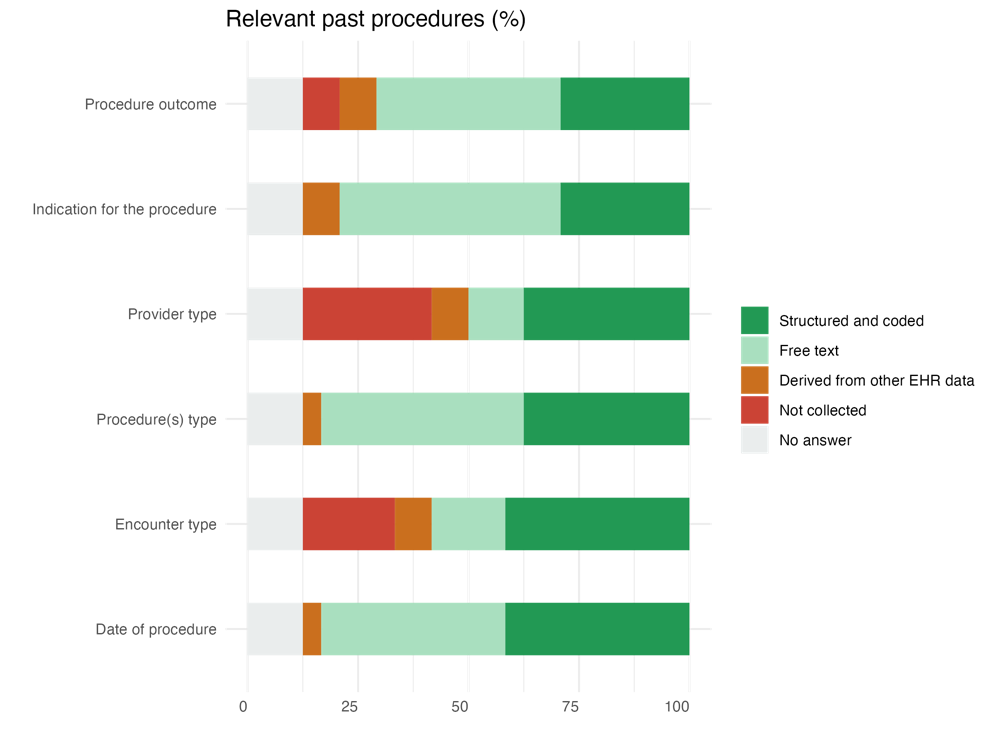


Figure 8: Percentage-stacked bar plot for relevant past procedures data items. Each colour represents the percentage of sites collecting data items in a particular format.

*Presence of other conditions*

Multiple birth history, developmental delay, and psychiatric disorders related to the patient were primarily captured in structured and coded formats (41.68% for development delay), though notable gaps were present. For instance, data on birth complications was structured at 56.67% of sites, leaving a significant proportion uncollected or derived from other sources. These inconsistencies in documentation may limit the holistic view of a patient’s medical background. Annex 1 explores these aspects further, and figure 9 displays the summarized data.


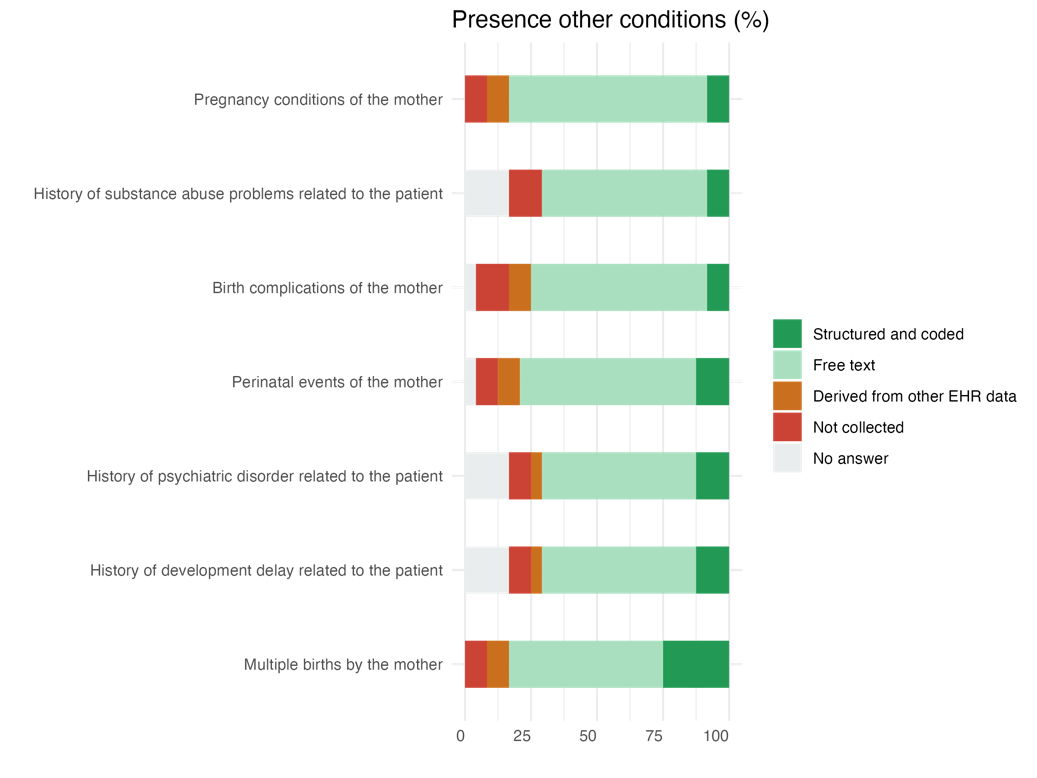


Figure 9: Percentage-stacked bar plot for presence of other conditions data items. Each colour represents the percentage of sites collecting data items in a particular format.

*Lifestyle and social status*

Data related to lifestyle factors like feeding type, caregiver education, and smoking exposure showed considerable variation in how sites captured the information. For example, personal smoking history was structured in only 58.33% of responses, with a significant proportion of data either uncollected or left unanswered. These inconsistencies may limit a comprehensive understanding of lifestyle factors influencing patient outcomes. For more in-depth data, see Annex 1, and figure 10 presents the main results.


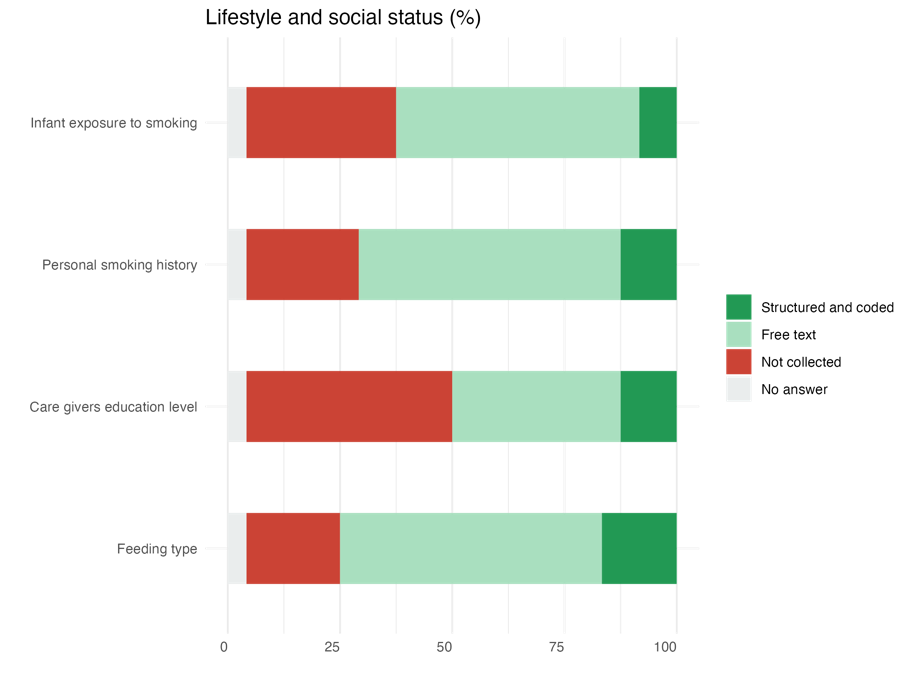


Figure 10: Percentage-stacked bar plot for lifestyle and social status data items. Each colour represents the percentage of each site collecting data items in a particular format.

*Allergies*

Collection of allergy data was highly variable. Hypersensitivity to medications was structured and coded at 50% of sites, and food allergies at 41.66%. However, a substantial proportion of data related to other allergies or confirmatory testing was uncollected or recorded as free text. This variability underscores challenges in tracking comprehensive allergy profiles across patients. Additional details can be found in Annex 1, while figure 11 summarizes these results.


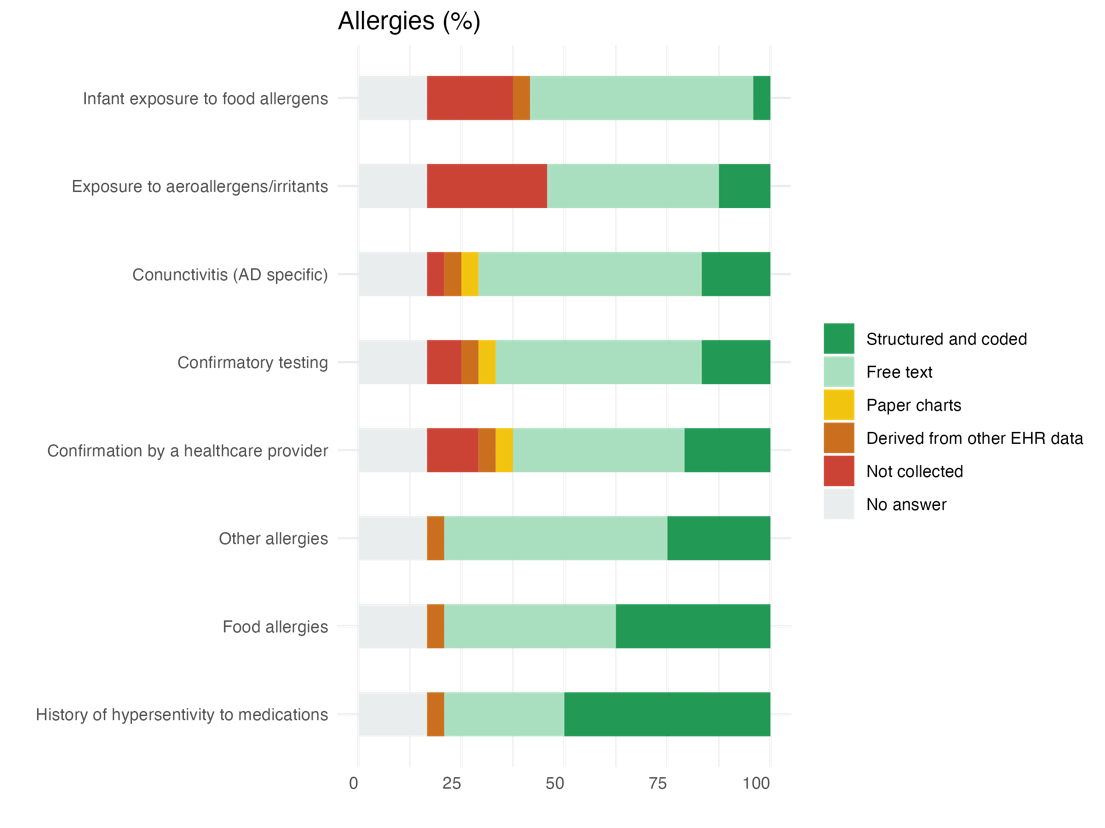


Figure 11: Percentage-stacked bar plot for allergy data items. Each colour represents the percentage of each site collecting data items in a particular format.

*Current findings*

Clinical measurements, such as weight and blood pressure, were consistently recorded in structured and coded formats, with systolic and diastolic blood pressure structured at 68.67% of sites. Temperature, heart rate, and oxygen saturation were similarly structured at over 60% of sites, providing a reliable source of vital patient metrics. However, a small number of sites continued to rely on paper-based or free-text entries, potentially complicating patient monitoring and longitudinal data analysis. Annex 1 provides further detail, and figure 12 displays a concise summary.


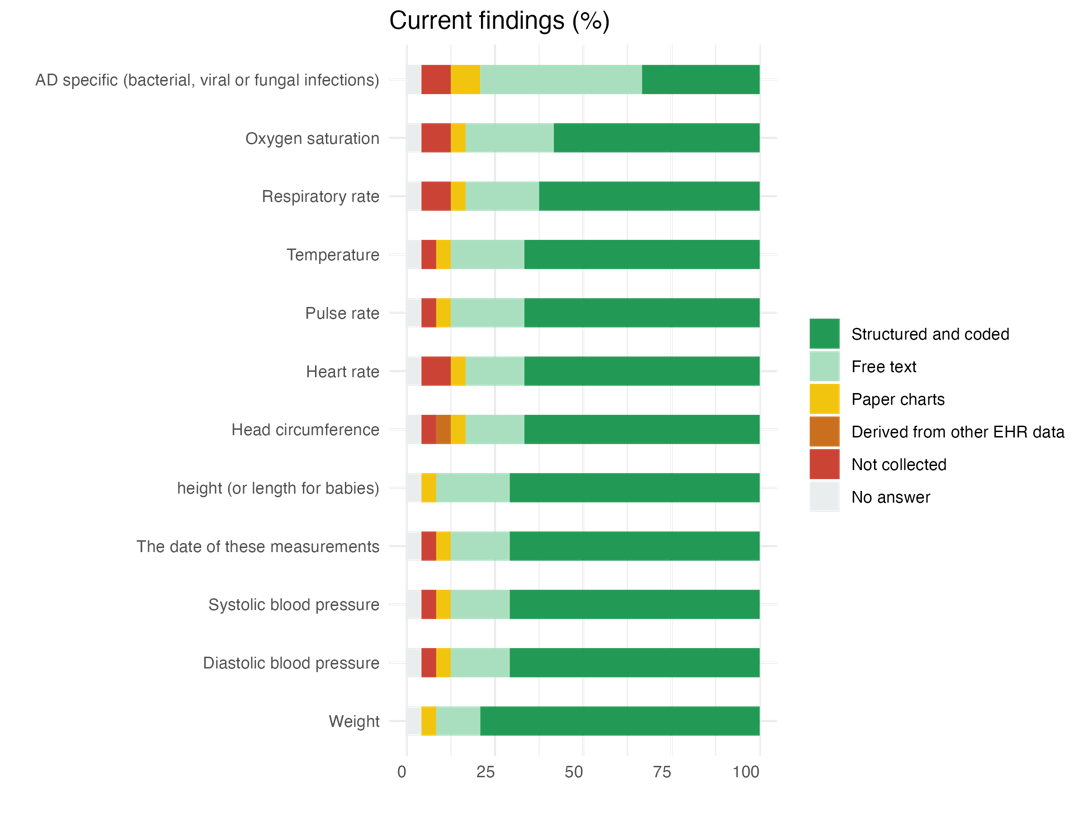


Figure 12: Percentage-stacked bar plot for current findings data items. Each colour represents the percentage of each site collecting data items in a particular format.

*Encounter History*

Dates of admission, discharge, and other encounter types were highly structured at most sites, with over 70% of responses capturing this data in coded formats. However, there were some gaps, with 16.67% of sites leaving these questions unanswered. Data related to referral sources and encounter type for primary care showed more variability, with only 50% of responses structured. These gaps may hinder a complete understanding of the patient's journey through the healthcare system. More information is provided in Annex 1, and figure 13 presents the key findings.


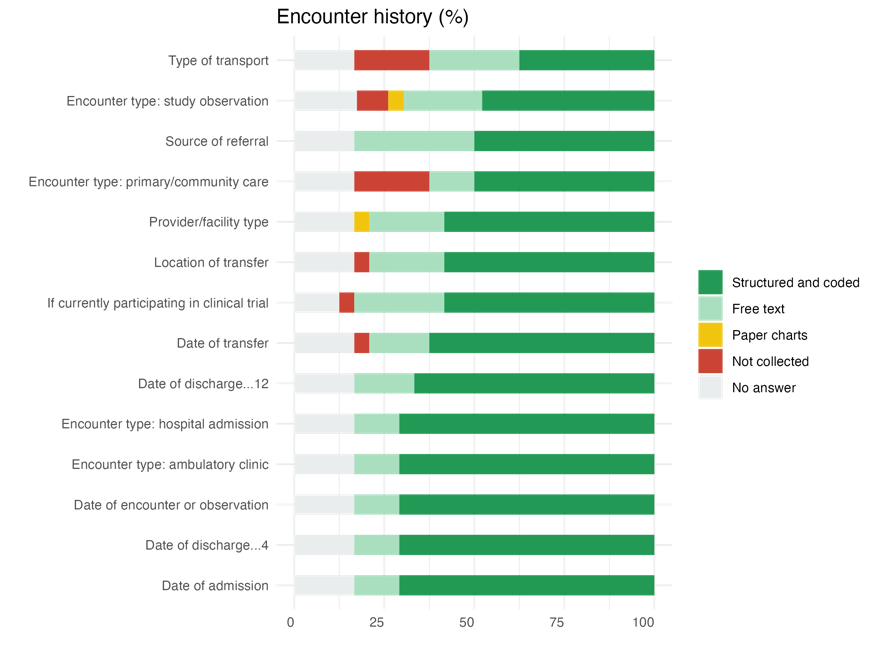


Figure 13: Percentage-stacked bar plot for encounter history data items. Each colour represents the percentage of each site collecting data items in a particular format.

*Radiotherapy: Neurofibromatosis (NF) specific*

Radiotherapy data related to NF was primarily structured and coded at most sites for key variables like dose (41.67%), frequency (37.49%), and start date (41.66%). However, a small proportion of data remained unstructured or uncollected, particularly regarding dose calculation methods and target volumes, which could impact standardized reporting of radiotherapy outcomes. Annex 1 provides further details, and Figure 14 shows a summary of the results.


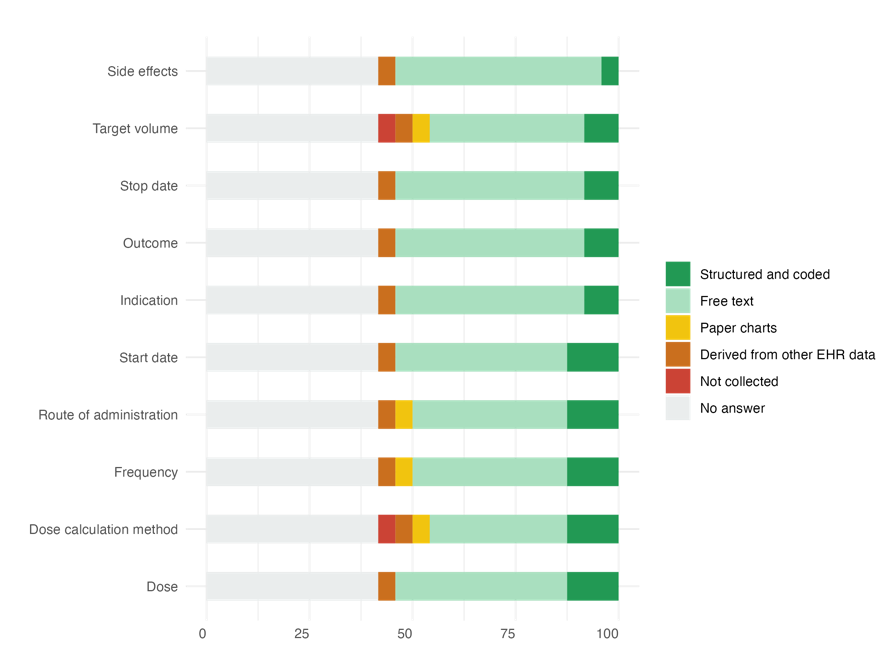


Figure 14: Percentage-stacked bar plot for radiotherapy (Neurofibromatosis specific) data items. Each colour represents the percentage of each site collecting data items in a particular format.

*Phototherapy Atopic dermatitis (AD) specific*

Phototherapy data, including dosage and frequency, was primarily captured in free-text formats (41.67%) with structured entries appearing in a minority of cases (16.67%). This variability in data capture, especially for key treatment indicators, may limit the study of phototherapy efficacy across sites. A more comprehensive analysis is available in Annex 1, and the summarized findings are presented in figure 15.


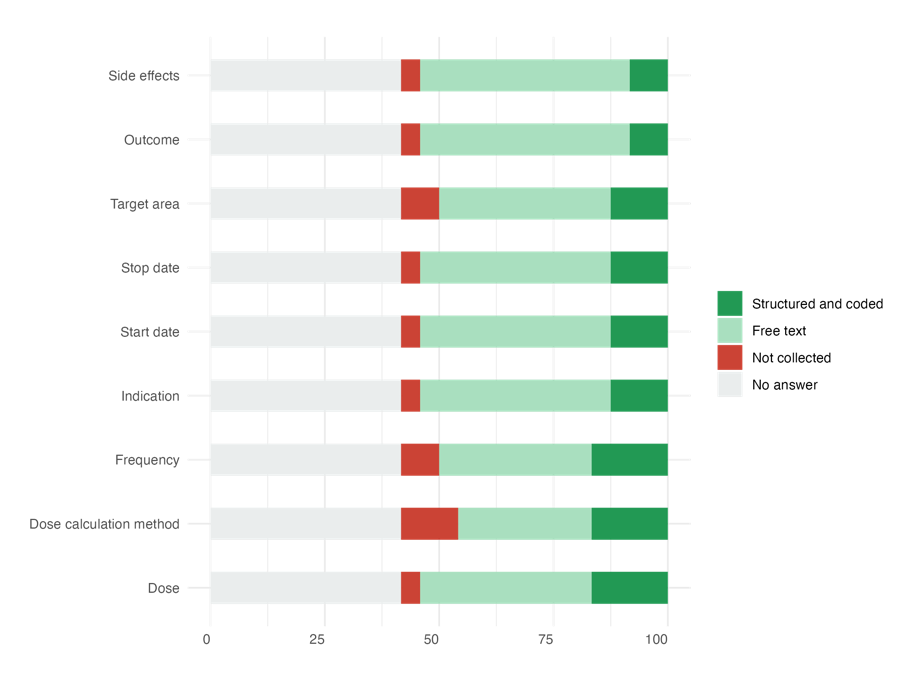


Figure 15: Percentage-stacked bar plot for phototherapy (Atopic Dermatitis specific) data items. Each colour represents the percentage of each site collecting data items in a particular format.
